# An orally available small molecule that targets soluble TNF to deliver anti-TNF biologic-like efficacy in rheumatoid arthritis

**DOI:** 10.3389/fphar.2022.1037983

**Published:** 2022-11-16

**Authors:** Alexander Vugler, James O’Connell, Mai Anh Nguyen, Dietmar Weitz, Thomas Leeuw, Elizabeth Hickford, Alexander Verbitsky, Xiaoyou Ying, Markus Rehberg, Bruce Carrington, Mark Merriman, Andrew Moss, Jean-Marie Nicholas, Phil Stanley, Sara Wright, Tim Bourne, Yann Foricher, Zhaoning Zhu, Daniel Brookings, Helen Horsley, Jag Heer, Laurent Schio, Matthias Herrmann, Srinivas Rao, Markus Kohlmann, Peter Florian

**Affiliations:** ^1^ Immunology Therapeutic Area, PV Early Solutions, UCB Pharma, Slough, United Kingdom; ^2^ Discovery Sciences, PV Early Solutions, UCB Pharma, Slough, United Kingdom; ^3^ Sanofi R&D, TMED Pharmacokinetics Dynamics and Metabolism, Frankfurt am Main, Germany; ^4^ Sanofi R&D, Drug Metabolism and Pharmacokinetics, Frankfurt am Main, Germany; ^5^ Sanofi R&D, Type 1/17 Immunology, Immunology & Inflammation Research TA, Frankfurt, Germany; ^6^ Development Science, PV Early Solutions, UCB Pharma, Slough, United Kingdom; ^7^ Sanofi R&D, Translation In vivo Models, Cambridge, MA, United States; ^8^ Sanofi R&D, Translational Disease Modelling, Frankfurt am Main, Germany; ^9^ Translational Medicine Immunology, PV Early Solutions, UCB Pharma, Slough, United Kingdom; ^10^ Development Science, Drug Metabolism and Pharmacokinetics, UCB Pharma, Braine-I’Alleud, Belgium; ^11^ Early PV Missions, PV Early Solutions, UCB Pharma, Slough, United Kingdom; ^12^ Milvuswood Consultancy, Penn, United Kingdom; ^13^ Sanofi R&D, Integrated Drug Discovery, Vitry-sur-Seine, France; ^14^ Global Chemistry, Discovery Sciences, PV Early Solutions, UCB Pharma, Slough, United Kingdom; ^15^ Sanofi R&D, Early Clinical Development, Therapeutic Area Immunology and Inflammation, Frankfurt am Main, Germany

**Keywords:** TNF, rheumatoid arthritis, small molecule, TNF trimer, immunogenicity

## Abstract

Tumor necrosis factor (TNF) is a pleiotropic cytokine belonging to a family of trimeric proteins with both proinflammatory and immunoregulatory functions. TNF is a key mediator in autoimmune diseases and during the last couple of decades several biologic drugs have delivered new therapeutic options for patients suffering from chronic autoimmune diseases such as rheumatoid arthritis and chronic inflammatory bowel disease. Attempts to design small molecule therapies directed to this cytokine have not led to approved products yet. Here we report the discovery and development of a potent small molecule inhibitor of TNF that was recently moved into phase 1 clinical trials. The molecule, SAR441566, stabilizes an asymmetrical form of the soluble TNF trimer, compromises downstream signaling and inhibits the functions of TNF *in vitro* and *in vivo*. With SAR441566 being studied in healthy volunteers we hope to deliver a more convenient orally bioavailable and effective treatment option for patients suffering with chronic autoimmune diseases compared to established biologic drugs targeting TNF.

## Introduction

TNF is one of the key cytokines involved in our rapid response to bacterial or inflammatory stimuli and drives the expression of other cytokines such as Interleukin-1 (IL-1) and Interleukin-6 (IL-6) (Fong et al., 1989; Apostolaki et al., 2010; Jang et al., 2021). It has both proinflammatory and immunoregulatory functions and is considered a pleiotropic cytokine (Vassalli, 1992; Kalliolias and Ivashkiv, 2016; Mehta et al., 2018). TNF is produced by immune cells such as macrophages and T/B cells as well as endothelial cells and mast cells and it is expressed as a membrane bound precursor (mTNF) that requires cleavage by TNFα converting enzyme (TACE, ADAM17) to release it as a soluble cytokine (sTNF) (Black et al., 1997; Moss et al., 1997, reviewed by Zunke and Rose-John, 2017). Both the soluble and transmembrane forms of TNF are biologically active in their trimeric forms (Jones et al., 1989) and signal through two separate receptors, tumor necrosis factor receptor 1 (TNFR1) and tumor necrosis factor receptor 2 (TNFR2). These receptors have clear differences in their cellular localization (Brockhaus et al., 1990; Hohmann et al., 1990, reviewed by Ward-Kavanagh et al., 2016; Gough, and Myles, 2020) with TNFR1 being ubiquitously expressed (Monaco et al., 2014) whilst TNFR2 expression is restricted to immune cells with higher expression being seen on Foxp3+ regulatory T cells (Treg) compared to naive CD4^+^ T cells (Chen and Oppenheim, 2011).

TNFR1 signaling uses the canonical nuclear factor-kappa B (NF-κB) and mitogen activated protein kinase (MAP kinase) pathway to deliver a proinflammatory signal (Tartaglia et al., 1993 reviewed by Ward-Kavanagh et al., 2016; Gough, and Myles, I., 2020). In contrast, TNFR2 signals *via* a non-canonical NF-κB pathway and activation of TNFR2 is important for proliferation, survival, and lineage stability of Treg cells, as well as the development of thymic Treg cells from Treg precursor cells (Chen et al., 2013; Mahmud et al., 2014; Salomon, 2021) and is associated with immunoregulation.

At physiological concentrations soluble TNF activates TNFR1 but not TNFR2, whereas the membrane bound form of TNF can activate both receptors (Grell et al., 1995). Uncontrolled production or function of TNF has been linked to the development of chronic inflammatory diseases (Alexopoulou et al., 1997; Lee et al., 2013) such as rheumatoid arthritis (RA), inflammatory bowel disease (IBD), psoriasis, psoriatic arthritis (PsA), ankylosing spondylitis and specific types of juvenile idiopathic arthritis (JIA) as was recently reviewed (Kalliolias and Ivashkiv, 2016).

TNF blockade by biologics has been a clinical success story (Willrich et al., 2015). These anti-TNF biologics are currently used successfully for the clinical treatment of RA, JIA, PsA, ankylosing spondylitis, psoriasis, and forms of IBD, particularly Crohn’s disease (CD) and ulcerative colitis (UC) (Mitoma et al., 2018).

RA is a common chronic inflammatory disease with a global prevalence of 0.51% (Almutairi et al., 2021). It is a complex autoimmune mediated disease that involves multiple inflammatory mediators such as TNF, IL-6 and IL-1 as well as immune cells (T cells, B cells, monocytes and macrophages) that help drive chronic inflammation of the joints (Cooles and Isaacs, 2011; Mellado et al., 2015). RA can lead to accumulating joint damage and irreversible disability (Smolen, 2018) but it can also result in extraarticular manifestations, such as rheumatoid nodules, pulmonary involvement or vasculitis, and other systemic comorbidities (Smolen et al., 2016). Today, the treatment options for RA are symptomatic agents, such as non-steroidal anti-inflammatory drugs (NSAIDs) and agents that target ‘disease modification’ that are termed disease-modifying antirheumatic drugs (DMARDs) which consist of both small molecules and biologics. Disease modification aims at improving the signs and symptoms of the disease for patients as well as normalizing physical function with an inhibition in the progression of structural damage to cartilage and bone.

The use of anti-TNF biologics such as infliximab, adalimumab, etanercept and certolizumab pegol have revolutionized the treatment of RA. Despite this success, the rate of disease remission in RA is still rather low with only 25% of patients reaching remission. In addition, it has also been reported that only 25% of patients achieve low disease activity (LDA) within 6 months (Smolen and Aletaha, 2015) of anti-TNF plus methotrexate treatment (Johnson et al., 2019). Additional limitations of the anti-TNF biologics include the development of adverse effects such as opportunistic infections, reactivation of latent tuberculosis and increased risk for specific malignancies, such as lymphomas (Kalliolias and Ivashkiv, 2016), as well as immunogenicity towards the drug itself resulting in the development of anti-drug-antibodies (ADA) that may limit their efficacy.

We believe that orally available small molecules have significant advantages over biologics, in terms of ease of administration, absence of immunogenic potential and production cost, particularly for chronic indications. The first significant breakthrough in the search for small molecule alternatives was described in 2005 (He et al., 2005). These inhibitors, exemplified by SPD-304, induced a disassembly of trimeric TNF that inhibited TNF signaling as shown in biochemical and cell-based assays. Less toxic analogs of SPD-304 were later described by Alexiou et al. (2014) and the same group reported improved molecules the following year (Papaneophytou et al., 2015). Attempts to build on this work are described by Deng et al. (2018) who reported a series of dihydrobenzoindole-6-sulfonamide inhibitors that occupy similar space to SPD-304. *In silico* tools were also used to identify plant-derived natural products that prevent the formation of TNF trimers (Papadopoulou et al., 2021). By applying virtual screening technology based on SPD-304, new inhibitors of the TNF-TNFR1 interaction have been identified (Sun et al., 2020) and recently, SPD-304 was used as a tool to discover molecules such as kaempferol from a natural product enriched DNA-encoded library (nDEL) (Wang et al., 2022).

The detailed mechanism of TNF signaling by TNF receptors is not fully understood. Nevertheless, there is evidence that receptor clustering is involved. Moreover, TNFR1 is thought to be present as a dimer held together by the so-called pre-ligand assembly domain (PLAD) (Chan et al., 2000; Deng et al., 2005; Deng et al., 2010). Crystallography shows that each TNF can bind three dimers of TNFR1, allowing one receptor from each dimer to bind a second TNF, since the binding site is facing outwards. This could in turn generate a larger signaling network comprised of TNF and its receptors (McMillan et al., 2021). It is likely that the disruption of such a signaling network by blocking one or more receptors from binding would impact TNF signaling. We have recently described several compounds (such as UCB-6786, UCB-5307 and UCB-9260) that do exactly this by stabilizing a distorted TNF trimer capable of binding TNFR1 at only two out of a possible three receptor binding sites (O’Connell et al., 2019). Furthermore, we demonstrated by thermal melt binding that these molecules are selective for TNF over other family members. Since the work described by O’Connell et al. was published, other groups have reported on the concept of stabilizing an asymmetric population of the TNF trimer. For example, an isoquinoline series was identified that was efficacious in a glucose-6-phosphate isomerase (GPI)-induced mouse arthritis model (Dietrich et al., 2021). In another study, a scaffold hopping approach based on the structures described by O’Connell et al. was applied to study a structure-activity relationship of quinolines and 1,5-naphthyridine TNF inhibitors (Xiao et al., 2020). A lead was identified that showed anti-TNF antibody-like efficacy in a collagen antibody induced arthritis (CAIA) model. Recent comprehensive reviews of this class of TNF inhibitor can be found in Medicinal Chemistry Reviews (Edmunds, 2019) and Drug Discovery today (Dömling and Xin, 2021). Despite the recent activity in small molecule targeting of TNF there has been no reported progression into clinical trials.

SAR441566, is a novel small molecule arising directly from the molecules first described by O’Connell et al., in 2019. The potency of SAR441566 was determined by protein and cell binding and activity against endogenous TNF in whole blood, while *in vivo* efficacy was assessed in an animal model of arthritis. This data, along with predictions from Quantitative Systems Pharmacology (QSP) and the molecule’s ADME and PK properties, were used to determine suitable doses for human clinical trials. Additionally, in order to provide a biomarker of TNF occupancy in humans, we developed an ELISA using an antibody described by Lightwood et al. (2021) that only detects compound-bound TNF.

Here we discuss the preclinical development of SAR441566, which has recently become the first anti-TNF small molecule to enter phase 1 clinical trials.

## Materials and methods

### Surface plasmon resonance binding of SAR441566 to tumor necrosis factor

SAR441566 binding to human TNF was demonstrated by surface plasmon resonance (SPR) using a Biacore T200 (GE Healthcare Bio-Sciences AB, Sweden). Here, the ligand TNF, was captured on the immobilized surface while the analyte SAR441566, was flowed over the captured surface. The sensor detects the change in mass on the sensor surface as the analyte binds to the ligand to form a complex on the surface. This corresponds to the association process. The dissociation process is monitored when the analyte is replaced by buffer. Human TNF (VCID7359) was produced by Beryllium (now UCB) in a stock concentration of 4.98 mg/ml at pH 7.4 and stored at −80°C. A solution of human TNF for the immobilization was prepared by dilution of the stock solution at concentration of 4.98 mg/ml to 10 μg/ml using immobilization buffer (Na acetate 10 mM pH 5.5). Human TNF was immobilized on a CM5 Series S Sensor Chip *via* amine coupling chemistry to a level of ∼1,500 Response Units (RU). HBS-P (10 mM HEPES pH 7.4, 0.15 M NaCl, 0.005% Surfactant P20, GE Healthcare Biosciences AB) was used as the running buffer with a flow rate of 10 μl/min. A reference surface was prepared by activating and deactivating an appropriate flow cell. SAR441566 was flowed over human TNF at five concentrations of doubling dilutions from 10 to 0.625 µM, plus a blank. The assay was run at 25°C with a flow rate of 100 μl/min in HBS-P supplemented with 1% dimethyl sulphoxide (DMSO). Double referenced background-subtracted binding curves were analyzed using the T200 BiaEvaluation software (version 1.0) following standard procedures. Kinetic constants for the on-rate (k_a_) and off-rate (k_d_) were determined for SAR441566 binding to human TNF from the fitting algorithm and the affinity constant determined (*K*_D_). The data were analyzed in BiaEvaluation software then presented in GraphPad Prism 7.

### Tumor necrosis factor potency using zymosan stimulated CD11b in human whole blood (WB assay)

The *in vitro* potency of SAR441566 was determined using an assay that measured zymosan-activated CD11b expression on granulocytes in human whole blood. Zymosan works by binding to toll-like receptor 2 (TLR2) and other receptors, including Dectin-2, (Sanguedolce et al., 1992), and has been shown to activate NF-κB signaling which induces the production of proinflammatory mediators, including TNF (Young et al., 2001; Zarkesh-Esfahani et al., 2004). In turn, TNF can stimulate the expression of CD11b (Mac-1, complement receptor 3), the α-subunit of the CD11b/CD18 heterodimeric complex (Montecucco et al., 2008) which can be measured by flow cytometry. Here a serial dilution (final assay concentrations 20–0.005 µM) of SAR441566 was pre-incubated with human whole blood (collected into BD vacutainer tubes containing ethylene diamine tetra acetic acid (EDTA), BD 367525) for 1 h 37°C/5% CO_2_ to allow uptake of the compound into cells. Zymosan (Invivogen, Tlrl-zyn), the assay stimulus, was added (final assay concentration 1 μg/ml in a total volume of 38 μl) with thorough mixing and incubated for 3 h at 37°C/5% CO_2_. The reactants were labelled by transferring 10 μl to a fresh assay plate containing 10 μl of anti-human CD45 antibody (Pacific Blue-labeled) and anti-human CD11b antibody (Phycoerythrin-labeled) for 1 h at 4°C. The blood was fixed/lysed overnight (4°C) using FACS/lysing solution (BD sciences, 349202) and analyzed with a FACS CANTO II (BD Biosciences). The number of single cells (SSC-A, SSC-W) positive for CD45 and expressing CD11b was determined using FlowJo software (BD Biosciences). Maximum inhibition of CD11b activation used for inhibition curve calculation was determined with an excess of an anti-TNF blocking antibody (in house anti-TNF antibody CB6). Activity Base (IBDS) was used to fit (4 PL) a dose dependent inhibition curve and calculate IC_50_ and E_max_.

### Tumor necrosis factor occupancy in human whole blood

An enzyme-linked immunosorbent assay (ELISA) was used to determine the potency at which SAR441566 occupies the TNF in zymosan activated human whole blood. For the TNF occupancy assay a serial dilution of SAR441566 was pre-incubated in 384 well plates with fresh human whole blood (collected in vacutainer tubes with EDTA, BD 367525) from UCB donors for 1 h at 37°C/5% CO_2_. Zymosan, was added (final assay concentration 1 μg/ml) with thorough mixing and incubated for 3 h at 37°C/5% CO_2_, as described for potency measurement. Plasma samples were collected by centrifugation at 2250g for 10 min and assayed for total TNF and TNF bound to SAR441566. The SAR441566 bound TNF was determined by ELISA using a capture antibody specific to compound-bound TNF (CA 1974), previously described in Lightwood et al. (2021), and a commercially available anti-TNF detection antibody (ab9348, Abcam). Total TNF was detected using a similar method in which the capture antibody was replaced with a commercially available anti-TNF reagent (MAB2101, R&D Systems). Standard curves for both ELISAs were prepared by serially diluting recombinant TNF (Beryllium/UCB) pre-complexed overnight with SAR441566, at concentrations of 10 μg/ml and 10 μM, respectively. Standards and samples were run in parallel in the two ELISAs and used to determine total TNF and SAR441566 bound TNF in the plasma samples. The ratio of SAR441566-bound TNF to total TNF was used to calculate percentage occupancy and plotted against SAR441566 concentration in the blood. A concentration-effect curve was generated to determine the concentration of SAR441566 which resulted in 50% occupancy (OCC_50_) and 90% occupancy (OCC_90_) of TNF using IDBS XLfit software.

Blood samples obtained from healthy donors at UCB Celltech were taken with informed consent under UCB Celltech United Kingdom HTA license number 12504. All human blood and blood products were then used, stored and disposed of in accordance with UCB’s internal procedures and the United Kingdom Human Tissue Authority (HTA) Act guidelines, Code E.

### Binding of SAR441566 to cell membrane tumor necrosis factor

The binding of SAR441566 to human TNF on the cell surface and intracellularly was assessed using an in-house non-secreting murine melanoma cell line (NSO) engineered to express a non-cleavable form of human membrane TNF (NSO-mTNFΔ1-12) (as described for murine TNF by Alexopoulou et al., 1997). Under sterile cell culture conditions, 1 ml of NSO-mTNF cells (non-adherent) were suspended at 1 × 106 cells/ml in 5 ml polystyrene round bottomed tubes (352052, Corning) in DMEM (Dulbecco’s Modified Eagle Medium, 21969-035, Gibco) containing 10% FBS (Fetal Bovine Serum, 10100147, Gibco) and ×1 NEAA (Non-Essential Amino Acid Solution, 11140050, Gibco). For both live cell and fixed and permeabilized cell experiments, SAR441566 was formulated and added to media containing NSO-mTNF cells at a final concentration of 1 μM and incubated for 60 min at 37°C. To measure the binding of SAR441566 to intracellular human TNF, cells were fixed and permeabilized using the FIX & PERM cell fixation & permeabilization Kit (ThermoFisher, GAS004) according to the manufacturer’s instructions. Antibody CA 1974, that recognizes the specific conformation of TNF when bound to SAR441566, was pre-labelled with Alexa Fluor^®^ 488 (AF488) using a Protein Labelling Kit (ThermoFisher, A10235) according to the manufacturers’ instructions and added to the fresh NSO-mTNF cells or fixed and permeabilized cells at a final concentration of 4 ng/ml (Room temperature, 60 min). Cells were plated into 96-well dark walled imaging plates (BD Biosciences, United Kingdom). Images were acquired using a Cellomics Arrayscan (Life Technologies) with a ×20 objective, ORCA-ER camera (Hamamatsu). Permeabilization of the cells allows CA1974 to enter the cell and bind intracellularly to the SAR441566 bound TNF complex while the use of live cells only captures SAR441566 bound to TNF on the cell surface.

### The binding kinetics of SAR441566 to membrane tumor necrosis factor on the cell surface

Binding kinetics was measured using NSO-mTNFΔ_1-12_ cells. Under sterile cell culture conditions, 1 × 10^6^ of NSO-mTNF cells (non-adherent) were suspended in 0.9 ml DMEM (Dulbecco’s Modified Eagle Medium, 21969-035, Gibco) containing 10% FBS (Fetal Bovine Serum, 10100147, Gibco) and 1x NEAA (Non-Essential Amino Acid Solution, 11140050, Gibco) in 5 ml polystyrene round bottomed tubes (352052, Corning). The final assay concentration of SAR441566 was 0.1 μM. This was formulated in identical assay media at ×10 final assay concentration (1 μM). To assess the binding kinetics of SAR441566, 100 μl of media containing 1 μM SAR441566 was added to the NSO-mTNF cells in 0.9 ml of media to give a final assay concentration of 0.1 μM SAR441566 in 1 × 10^6^ of NSO-mTNF cells. Importantly, SAR441566 and CA 1974 (4 ng/ml) were added at the same time (Time 0). The increase in AF488 labelling of the cells reflects the increase in the changed conformation of TNF bound to SAR441566. Cells were analyzed and recordings taken at baseline and multiple time points up to 90 min by flow cytometry (FITC/time, FACS Carousel), gated by FSC, SSC, and AF488 staining. Mean fluorescence intensity increased over time and was measured and used to calculate the kinetics of binding and populate the QSP model predictions (Figure 5B).

This protocol is applicable to all small molecules in this class (Figure 5B). Antibody CA1974 recognizes TNF bound to all compounds that stabilize asymmetric TNF, having been raised against TNF bound to UCB-9260 (Lightwood et al., 2021).

### *In vivo* efficacy of SAR441566

*In vivo* efficacy was assessed in the collagen-induced arthritis (CIA) model in male DBA/1 mice, across three independent studies. All *in vivo* studies were reviewed by an internal Ethical Review Body and conducted in accordance with the Animals (Scientific Procedures) Act 1986, EU Directive 2010/63/EU. Chicken type II collagen (CII, MD Biosciences) was dissolved in 0.1 M acetic acid at a concentration of 2 mg/ml and rolled overnight at 4°C. The next day (Day 0) the CII solution was emulsified in an equal volume of complete Freund’s adjuvant (Sigma Aldrich) to give a final concentration of 1 mg/ml. Mice aged 10–15 weeks old were sensitized with 0.1 ml (100 µg CII) of emulsion injected intradermally on the flank under anesthesia. Fourteen days post-sensitization mice received a booster intradermal injection of CII emulsified in incomplete Freund’s adjuvant (Sigma Aldrich) on the opposite flank. Mice were assessed for signs of arthritis from Day 14 onwards. When signs of arthritis were seen, mice were weighed, and disease scored. Disease score was graded on a 0 to 3 scale as follows: grade 0, no swelling; grade 1, wrist or ankle swollen; grade 2, wrist/ankle and pad swollen; grade 3, wrist/ankle, pad and digits swollen. Disease score was the sum of all 4 paws (maximum score per mouse was 12). Mice were dosed therapeutically for 20 days with either vehicle or SAR441566 (100 µL orally twice a day (BID)) starting from the first day they showed signs of arthritis. The efficacy of SAR441566 was assessed over a dose range of 1, 3, 10 and 30 mg/kg. The area under the curve (AUC) of the disease score was calculated from baseline for individual mice in each treatment group. Blood samples were taken *via* the tail vein from a subset of mice in each group at 1 and 14 h post dose of SAR441566 on day 1 to obtain exposure levels.

In one of the CIA studies an in-house anti-mouse TNF antibody (Ab501), that has previously been shown to be efficacious in an arthritis model (O’Connell et al., 2019), was used as a positive control. Ab501 was dosed intravenously at 100 mg/kg on the first day mice showed signs of arthritis followed by subcutaneous administration at 100 mg/kg twice weekly. The limb bones from this study were used for the micro-CT analysis described below and the efficacy data from the three individual CIA studies are shown in the Supplementary Figures 2A–F.

### *In vivo* data analysis

The mean area under the curve (AUC) change from baseline data was analyzed using a linear model suitable for the randomized design. The statistical analysis was performed in a Bayesian framework (Walley et al., 2016) fitting the linear model with vague priors for treatment groups (∼N (0, 1e^4^)) and assuming a normal distribution with a vague prior for the variance between observations (∼Gamma (1e^−3^,1e^−3^)) (Gelman et al., 2004). The posterior distributions of the percentage reductions of each dose group from the vehicle group were used to make probability statements about effects of scientific interest.

### Micro-computed tomography imaging

X-ray micro-computed tomography (micro-CT) imaging of mouse limbs originating from the CIA model was performed using a SkyScan 1176 scanner (Bruker, Kontich, Belgium). Dissected mouse limbs of each animal were fixed in 10% neutral buffered formalin. One day before micro-CT imaging, limb bone samples were carefully washed in phosphate-buffered saline (PBS) and then installed in polypropylene syringes, one for each with labels, at 4°C with 35% ethanol.

Prior to micro-CT scanning, samples were warmed to room temperature. A batch scanning approach (Ying et al., 2017; Shazeeb et al., 2018) with improved scanning resolution was used for the micro-CT imaging of all limb bones, in which each 18 sealed syringes of limb bones were scanned together in a batch with another syringe containing 0.25 and 0.75 g/cm^3^ calcium hydroxyapatite phantoms. Scanning settings of 50 kV, 500 μA, 8.6 µm pixel resolution, 0.15° or 0.3° rotation steps, and 4-frame imaging average with a 0.5-mm Al filter were applied.

The acquired X-ray projection images were reconstructed using NRecon software (V1.7.3, Bruker) with beam hardening correction and post-alignment. Three-dimensional (3D) bone images of limbs were segmented and recorded individually with their labels for analysis.

### 3D volumetric micro-CT bone image analysis

For 3D micro-CT analysis of the tibia trabecular, cortical, and whole bones, MATLAB (V9.9, MathWorks, MA) protocols were developed to automatically select volume of interest (VOI) for each limb bones. CT-Analyzer (CTAn, V1.18, Bruker) was then used to segment and analyze the limb bones. After all, VOIs of micro-CT images were obtained, automatic batch processing was developed and applied for two-dimensional (2D) section analysis and 3D volumetric analysis, including bone mineral density (BMD) calculation. This analysis was applied to assess limb paws with 400-slice VOIs. The trabecular bone analysis methods and parameters (Skyscan, 2009) were also used for whole bone microstructure analysis. Amira software (V6.1.1, Thermo Scientific, MA) was used to visualize the reconstructed digital bones and the segmented VOIs, in 3D rendering and x-y, x-z, y-z views, for 3D imaging data validation. Statistical analysis was performed using GraphPad Prism 9.0 (GraphPad Software, CA).

### *In vitro* ADME and *in vivo* PK

The permeability of SAR441566 was determined at pH 7.4 in trans-well experiments with Caco-2 TC7 cells, internally validated for BCS classification (ICH-M9) with the probe substrates listed in the guideline. Every week cells are controlled for monolayer integrity using mannitol and low and high permeable compounds (metoprolol and propranolol) with acceptance criteria. In addition, digoxin is used for P-gp positive control in the absence and presence of cyclosporine A. Bi-directional flux/efflux experiments with increasing concentrations of SAR441566 and specific inhibitors for P-gp and BCRP (1 µM PSC833 purchased from Tocris and 1 µM Ko-134 purchased from Solvo, respectively) were used to characterize efflux transporter substrate properties of SAR441566. Fraction unbound of SAR441566 across preclinical species and human was determined by equilibrium dialysis with [3H]-labelled SAR441566 at six concentrations between 0.01 and 50 μg/ml at 37°C for 2 h, where equilibrium between plasma and buffer was reached. *In vitro* metabolic clearance was determined in cryopreserved primary human hepatocytes (20-donor mixed gender pool obtained from BioIVT). The incubation was performed using suspended cells as described in Ménochet et al. (2012). This assay is routine, performed using commercially available cells and did not require specific review by an ethical committee. *In vivo* PK studies were performed after intravenous (i.v.) and oral (p.o.) administration in rat, dog and cynomolgus monkeys at various doses using recommended application volumes for the different species: Rat 1 ml/kg for IV, 2 or 10 ml/kg for PO, dog 0.3 ml/kg IV, 5 ml/kg PO, Cynomolgus Macaque 2 ml/kg IV and 5 ml/kg PO. Studies were carried out according to internal guidelines and governmental requirements. SAR441566 was quantified from EDTA plasma and tissue homogenates. Tissue samples were weighed, homogenized in water and diluted in rat plasma with an exploratory liquid chromatography-mass spectrometry (LC-MS/MS) method after protein precipitation, with a quantification limit between 1 and 100 ng/ml. The pharmacokinetic parameters were calculated by the program Phoenix WinNonlin 6.4 using a non-compartmental model and linear trapezoidal interpolation calculation.

### Quantitative systems pharmacology model predictions on tumor necrosis factor reduction in synovial tissue

We adapted a published dynamic, semi-mechanistic model on the TNF (trimer) biology (Fallahi-Sichani et al., 2011) to the conditions of the synovial tissue using additional data on cell numbers, cytokine levels (Chappard et al., 2001) and binding kinetics (Cope et al., 1992; Grell et al., 1998; McNearney et al., 2004) in the SimBiology^®^ software (using MATLAB^®^ R2020b). Changes to nominal parameters to this model are within the bounds provided by the source. Only intracellular TNF production, binding by SAR441566 and trafficking to the cell membrane was added to the model, which is based on the TNF residence time of 2–3 h measured in the membrane binding assay (Figure 5B). To model assay data on TNF-Small molecule (SM) complex appearance on cell surfaces, binding of the mTNF-SM complex by a staining antibody and disappearance of that complex was also added as model variant. To assess the prediction uncertainty, we sampled 100 parameter sets from within the parameter bounds, followed by simulation of the percent TNF inhibition.

### Human PK prediction

PK studies in rat, dog and cynomolgus monkey, were performed to determine human clearance and volume of distribution (Vss) by simple three-species allometry with correction for interspecies differences in fraction unbound in plasma (Mahmood and Balian, 1996). In addition, human clearance and Vss were estimated using mechanistic models based on *in vitro* data. Total clearance was assumed to be the sum of hepatic and renal clearance, with renal clearance being negligible as only traces of unchanged compound could be recovered in a mass balance study in rats (data not shown). Scaling hepatic clearance from *in vitro* to *in vivo* was performed using a value of 8 μl/min/10^6^ cells for *in vitro* intrinsic clearance as determined in suspended human hepatocytes (Table 2) using the well-stirred liver model with or without correction for albumin binding, respectively, as described in the literature (Poulin et al., 2012). Vss was calculated based on pKa, logP and plasma protein binding (see Table 2) using the Rodgers and Rowland method as incorporated in the Simcyp^®^ Simulator V16R1 (Method 2) (Rodgers and Rowland, 2007).

## Results

### SAR441566 binds with high affinity to human tumor necrosis factor by surface plasmon resonance

**FIGURE 1:**
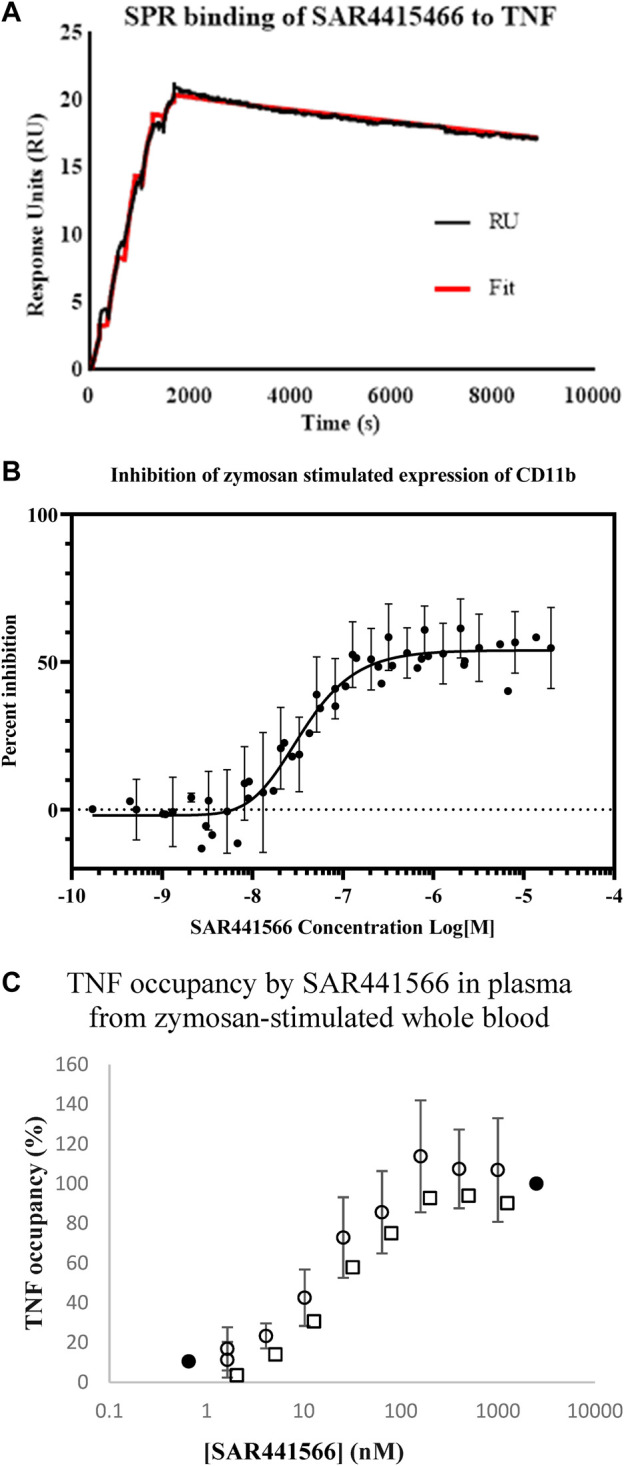
*In vitro* analysis of SAR441566: **(A)** shows a representative Biacore T200 curve for SAR441566 binding to TNF immobilized on a CM5 sensor chip. The KD was determined to be 15.1 nM (n = 4) (Table 1). **(B)** inhibition of zymosan stimulated expression of CD11b on granulocytes in human whole blood by total (free and protein bound) SAR441566 with an IC50 of 35 nM (n = 50) (Supplementary Figures S1A,B show a representative CD11b inhibition curve and corresponding flow cytometry analysis. **(A)** TNF occupancy in plasma samples from zymosan-stimulated whole blood, was determined from the ratio of compound-bound to total TNF using the conformation specific and total TNF ELISAs (seven individual blood donors assessed in seven independent experiments). Blood from four donors was spiked with SAR441566 concentrations ranging from 1.6–1,000 ng/mL, presented as open circles on the graph. Blood from one donor was spiked with an extended curve including the additional concentrations of 0.66 and 2500 ng/mL (closed circles). Mean occupancy +/− standard deviation plotted for SAR441566 in the range of 1.6–1000 ng/mL for the total of these five donors. Blood from two further donors was spiked with SAR441566 in the range of 2.0–1250 ng/mL (open squares). TNF occupancy was proportional to SAR441566 concentration and plateaued at around 100%, indicating full occupancy.

The affinity of SAR441566 for human TNF was determined using SPR technology on a Biacore T200 (Table 1 and Figure 1A). The study showed that SAR441566 bound to human TNF with an average affinity (dissociation constant [K_D_]) value of 15.1 nM (*n* = 4) (Table 1). The average association rate constants and dissociation rate constants for SAR441566 binding to human TNF are 1,471 (M^−1^s^−1^) and 2.19e^−5^ (s^−1^), respectively. The average t_1/2_ of SAR441566 for human TNF was determined to be 9 h:

**TABLE 1:** Affinity of SAR441566 binding to human TNF.

SAR441566	k_a_ (M^−1^s^−1^)	k_d_ (s^−1^)	t_1/2_ (h)	K_D_ (nM)
Human TNF	1,325	2.53e^−5^	7.6	19.1
Human TNF	1,579	1.71e^−5^	11.3	10.8
Human TNF	1,565	2.02e^−5^	9.5	12.9
Human TNF	1,414	2.49e^−5^	7.7	17.6
**Average**	**1,471**	**2.19e** ^**−5**^	**9.0**	**15.1**
STD	123	3.93e^−6^	1.7	3.9

K_D_: dissociation constant; k_a_-association rate constant; k_d_-dissociation rate constant; t_1/2_: half-life.

### SAR441566 potently inhibits zymosan-activated CD11b expression on granulocytes in human whole blood

The potency of TNF inhibition by SAR441566 was assessed in a human whole blood assay. Endogenous TNF expression is driven by Zymosan and this in turn produces CD11b on the cell surface.

Briefly, the potency of SAR441566 to inhibit TNF in human whole blood was assessed by the addition of serial dilutions of the compound to blood prior to the addition of zymosan and, following an incubation, detection of CD11b expression on granulocytes by flow cytometry.

SAR441566 inhibited the expression of CD11b in zymosan-stimulated human whole blood with an IC_50_ of 35 nM (geometric mean; n = 50, 95% CL, 27.7–44 nM) and an IC_90_ of 163 nM (geometric mean; 95% CL, 139–191 nM) (Figure 1B). This value represents the inhibition by the total amount of SAR441566 rather than the ‘free’ non-protein bound compound.

### SAR441566 fully occupies human tumor necrosis factor

In addition to measuring inhibition of endogenous TNF in a CD11b assay, we also determined the potency at which SAR441566 occupies TNF in human whole blood. Whole blood from seven individual donors was incubated in the presence of serial dilutions of SAR441566, giving final assay concentrations of between 0.1 nM and 2.5 µM, and stimulated by zymosan. The ratio of total TNF and SAR441566-bound TNF was determined by ELISA using antibodies that detect total TNF and the conformational selective antibody (CA 1974) to detect compound bound TNF. TNF occupancy increased proportionally with SAR441566 concentration and concentration-response curves were generated to determine the concentrations of SAR441566 required to achieve 50% occupancy (OCC_50_) and 90% occupancy (OCC_90_) (Figure 1C).

SAR441566 occupied human TNF with an OCC_50_ for total compound (includes protein bound) of 14.3 nM; (geometric mean; *n* = 12, 95% CL, 9.7 nM- 21.2 nM), and with an OCC_90_ of 64.3 nM (geometric mean; n = 12, 95% CL, 36.5 nM–113.1 nM). This equates to a free SAR441566 OCC_90_ concentration of 16 nM.

### ADME properties and intracellular binding to human tumor necrosis factor

SAR441566 is a small molecule drug with a molecular weight of 502.5 Da, a moderate hydrophobicity and a partially charged tertiary amine with a pKa of 8.6. The molecule does not violate any of the 5 rules of Lipinski (Lipinski et al., 1997). SAR441566 showed a low metabolic clearance in human hepatocytes and a high fraction unbound of 25% in human plasma with a blood/plasma (B/P) ratio of 1 (Table 2).

**TABLE 2:** Physicochemical and *in vitro* ADME properties.

Molecular weight (g/Mol)	502.5	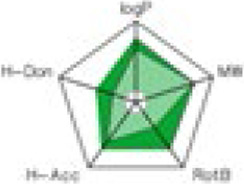	Permeability^a^	High
logP^c^	1.74		Clearance^b^	8.0
H-Donor	2		Fraction unbound human plasma	25%
H-Acceptor	8			
Rotatable Bonds	4		B/P ratio	1
PSA (A^2^)	99.2			
Lipinski Ro5: MW < 500, LogP < 5, H-Donor < 5, H-Acceptor < 10, Rotatable Bonds < 10, PSA < 140 A2 (Lipinski et al., 1997)				

^a^ Caco-2 TC7 cells validated for BCS, classification (Simkin and Bassett, 2011), in the presence of a P-gp inhibitor.

^b^ Suspended human hepatocytes µl/min/10^6 cells.

^c^ in silico prediction.

In Caco-2 TC7 cells, absorption was pH- and concentration-dependent with a higher absorption at a neutral pH of 7.4 compared to pH 6.5. At saturating concentrations SAR441566 was a high permeable compound. Bi-directional Flux/Efflux experiments identified SAR441566 as a substrate of efflux transporters and further analysis with specific inhibitors PSC833 (P-gp inhibitor) and Ko-143 (BCRP inhibitor) revealed SAR441566 as a P-gp substrate (Table 3).

**TABLE 3:** Transport characterization of SAR441566 in Caco-2 cells.

Condition (apical/basal)	SAR441566 concentration (μM)	Inhibitor (1 μM)	Flux (nMs^−1^)	Efflux (nMs^−1^)
BSA 0.5%/5% pH 6.5/7.4	20	—	15.3	—
BSA 0.5%/0.5% pH 7.4/7.4	20	—	46.9	64.8
BSA 0.5%/0.5% pH 7.4/7.4	1	—	7.43	208
BSA 0.5%/0.5% pH 7.4/7.4	1	PSC-833	74.1	109
BSA 0.5%/0.5% pH 7.4/7.4	1	Ko-134	8.96	203

In rat, a direct elimination of parent SAR441566 into the gut was observed in addition to oxidative metabolism in a mass balance study (data not shown).

As a highly permeable and partially cationic compound, SAR441566 showed the expected high tissue distribution in rat, with tissue to plasma ratios up to 53 with highest accumulation in kidney, lung and liver, and very low distribution into the brain (Tables 4, 5).

**TABLE 4:** Tissue Distribution of SAR441566 in rat (80 mg/kg PO).

Tissue	Tissue/Plasma ratio AUC_0-72_
Kidney	52.9
Liver	26.3
Lung	31.2
Brain	<1

**TABLE 5:** Pharmacokinetic parameters following single administration to male animals.

Species	Route	Dose (mg/kg)	t_max_ (h)	C_max_ (ng/ml)	AUC_inf_ (ng.h/ml)	CL (L/h/kg)	V_z_ (L/kg)	T_1/2,z_ (h)	Bioavail-ability (%)^a^
Rat	IV	1		424	731	1.38	4.1	2.1	
Rat	PO	3	4	73.6	573				26
Dog	IV	1		1,510	4,200	0.267	2.68	7.00	
Dog	PO	30	2	6,920	131 000				108^b^
Dog	PO	100	7	6,320	258 000				62^b^
Cyno	IV	1		774	2,970	0.355	2.59	6.66	
Cyno	PO	3	2	609	6,620				67^b^
Cyno	PO	10	6	1,250	20 000				68^b^
Cyno	PO	20	8	2,490	45 200				77^b^
Cyno	PO	100	6	3,640	107 000				36^b^

^a^ Bioavailability (%) = AUC (oral)/AUC (IV) * Dose (mg/kg) IV/Dose (mg/kg) oral.

^b^ I.v. and p.o. exposure values used for bioavailability calculation taken from different studies.

AUC_inf_: area under the plasma concentration-time curve from time zero to infinity; C_max_: maximum (peak) plasma drug concentration; CL: clearance; Cyno: cynomolgus monkey; t_max_: time to reach maximum (peak) plasma concentration following oral drug administration; t_1/2,z_: terminal half-life; V_z_: volume of distribution.

**FIGURE 2:**
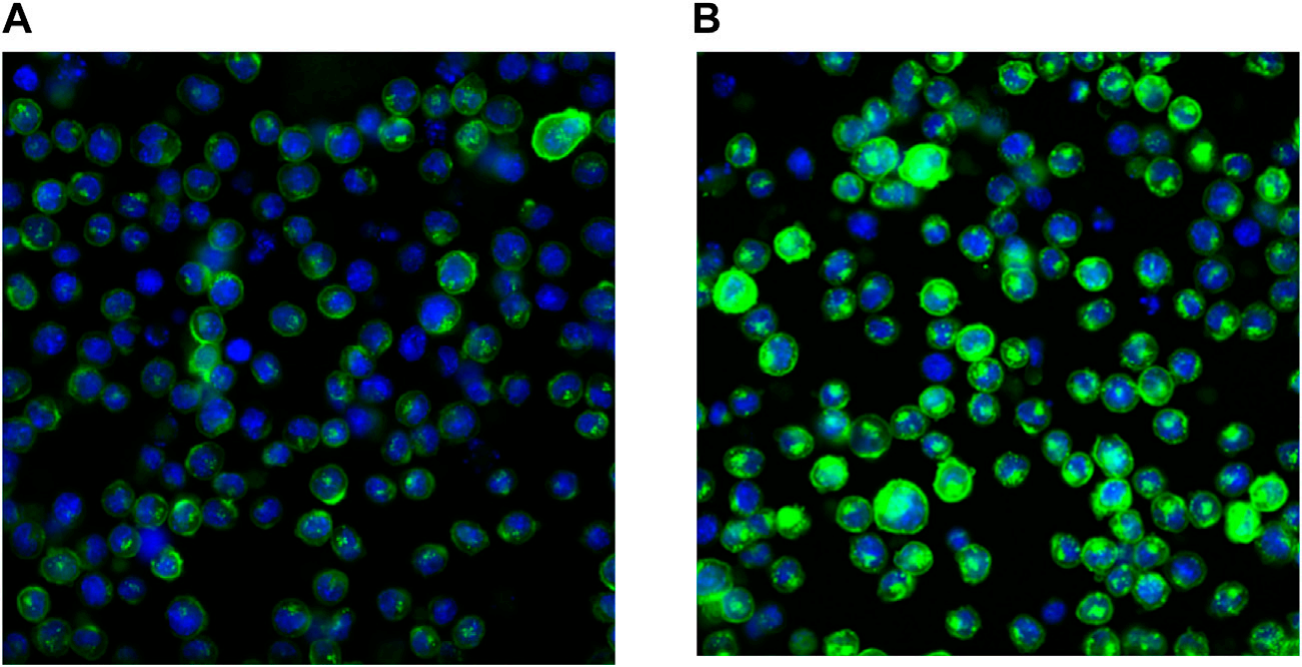
Binding of SAR441566 to human TNF on cells: The binding of SAR441566 to human TNF on the cell surface and intracellularly. An in-house NSO cell line expressing a non-cleavable form of membrane TNF was generated (NSO-tmTNFΔ_1-12_ Alexopoulou et al., 1997). An Alexa Fluor 488 labelled conformation specific antibody (CA1974) that only binds TNF when in complex with SAR441566 was used to determine binding on live cells **(A)** and intracellularly after cells were fixed and permeabilized **(B)**. Images were acquired using a Cellomics Arrayscan (Life Technologies) with a 20× Objective, ORCA-ER camera.

This behavior is related to the physico-chemical properties of SAR441566. It has been previously described for cationic drugs in general, as a consequence of unspecific binding to phospholipids and some lysosomal/endosomal storage because of the pH gradient between the cytosol and the intracellular compartment (Holt et al., 2019; Schmitt et al., 2019). The membrane form of TNF is stored in the acidic Golgi vesicles (Shurety et al., 2000) before it is transported to the plasma membrane where it may be shed to form soluble circulating TNF. As a consequence of its favorable distribution properties, SAR441566 is capable of binding to its target inside these vesicles, before the TNF is transported to the plasma membrane. We calculated a >150 fold accumulation in the vesicles at the target site using the method described in Schmitt et al. (2019). The accumulation rate will be further increased by the very slow off rate of SAR441566 from TNF as shown by SPR (Table 1). The binding of SAR441566 to human TNF intracellularly and on cellular membranes was assessed in an NSO cell line expressing a non-cleavable form of membrane TNF with Alexa Fluor^®^ 488 labelled IgG antibody (CA 1974) to detect TNF bound to SAR441566. Experiments allowed us to determine binding of SAR441566 to TNF on the plasma membrane of living cells (Figure 2A) as well as intracellularly after fixation and permeabilization of the plasma membrane (Figure 2B).

SAR441566 demonstrates ideal pharmacokinetic properties for the inhibition of TNF in blood and tissues. This is a consequence of good permeability, a low metabolic clearance, the high tissue distribution and a high fraction unbound, that improves tissue penetration, (in combination with the slow off rate from the target).

### SAR441566 demonstrates antibody-like efficacy in the collagen-induced arthritis (CIA) model

**FIGURE 3:**
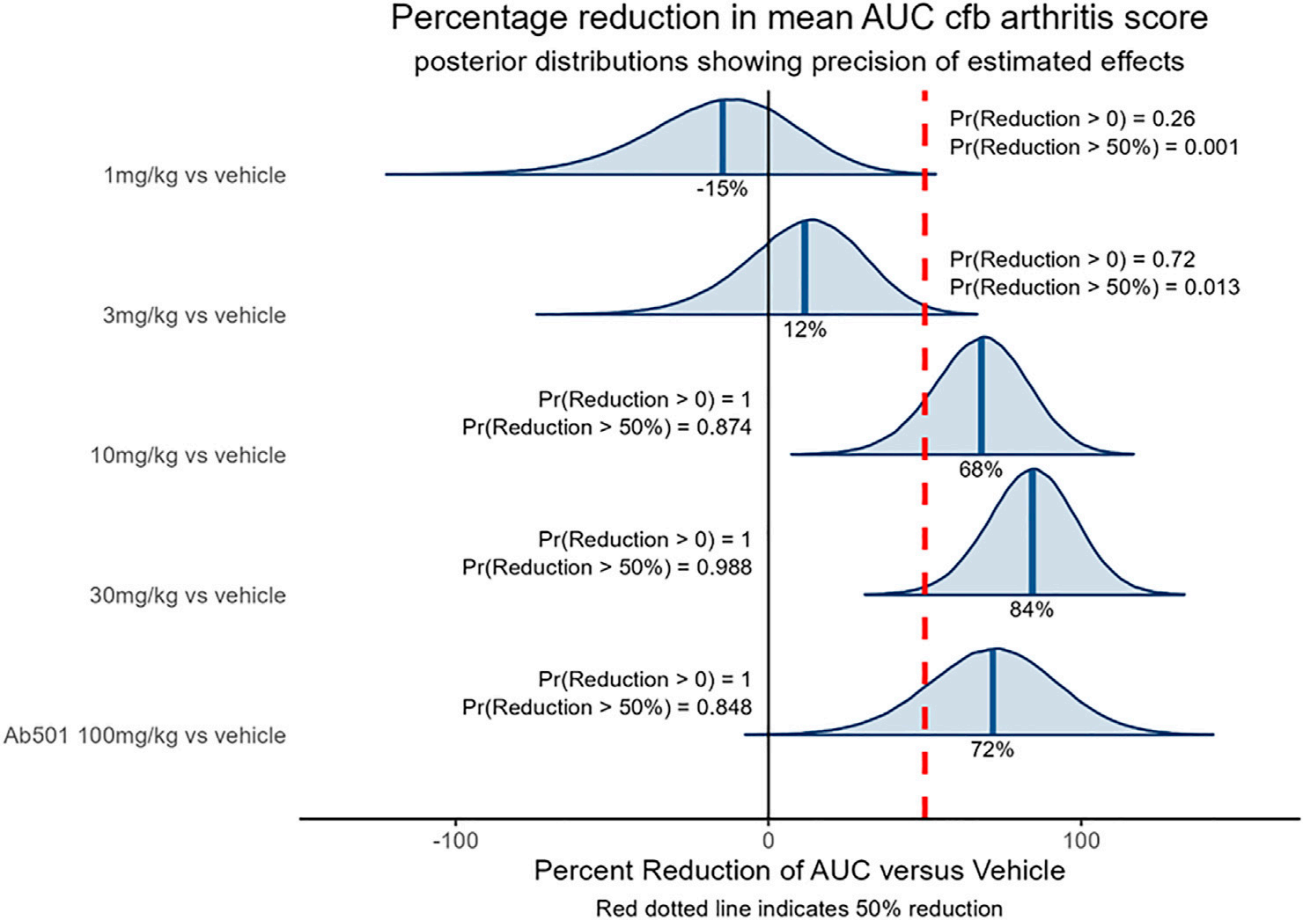
*In vivo* efficacy of SAR441566 in the CIA Model: The dose dependent *in vivo* efficacy of SAR441566 in the CIA model when dosed therapeutically. SAR441566 was dosed orally BID at 1, 3, 10 and 30 mg/kg. Mouse anti-TNF antibody (Ab501) was dosed at 100 mg/kg. Efficacy assessed by the ability of SAR441566 to reduce the mean area under the curve (AUC) change from baseline (cfb) arthritis score compared to vehicle treated mice. Percent inhibition of each group is indicated on the graph. Data analyzed using a linear model suitable for the randomized design. Red dotted line represents a 50% reduction in the AUC of the disease score. Probability (Pr) statements of scientific interest were made using Bayesian analysis. Graph shows combined data from 3 independent CIA studies. The data demonstrates that the *in vivo* efficacy of SAR441566 at 10 and 30 mg/kg is comparable to an anti-TNF biologic (Ab501). Vehicle treated group n = 50, SAR441566 1 mg/kg n = 17, 3 mg/kg n = 26, 10 mg/kg n = 31, 30 mg/kg n = 34 and Ab501 treated group n = 16.

The CIA model is a common *in vivo* model of rheumatoid arthritis used in drug discovery to identify anti-arthritic drugs. The model is induced by sensitizing mice to chicken type II collagen, as described previously (Brand et al., 2007), which in turn stimulates a humoral and cellular immune response that manifests in the joints as an inflammatory arthritis. The model is responsive to clinically used anti-arthritic therapies (Bendele et al., 1999) including biological agents directed against TNF (Wang et al., 2013) therefore demonstrating its clinical relevance. The ability of SAR441566 to inhibit arthritis progression in the CIA model was assessed therapeutically over a dose response curve from 1 to 30 mg/kg across three independent studies. The combined data set clearly shows that oral administration of SAR441566 results in a dose dependent inhibition in signs of arthritis. SAR441566 dosed at 10 mg/kg resulted in a 68% reduction in the AUC of the disease score compared to vehicle treated mice and this inhibitory effect increased to 84% at a dose of 30 mg/kg (Figure 3). Whilst we could not compare SAR441566 directly with marketed biologics as they are specific to human TNF or have a different affinity for mouse, the inhibitory effect of SAR441566 at both 10 and 30 mg/kg was comparable to that seen with an anti-mouse TNF antibody (Ab501) which reduced AUC of the disease score by 72%. Using Bayesian analysis, probability statements of scientific interest can be made and in Figure 3 the red dotted line represents a 50% reduction in the AUC of the disease score. SAR441566 at a dose of 10 mg/kg has a probability of 0.87 that it will reduce the AUC of the disease score by more than 50% while at 30 mg/kg there is a 0.99 probability it will reduce the AUC by 50%. Again, this was comparable to Ab501 which has a probability of 0.85 of reducing the AUC by 50%. These data demonstrate that at a dose of 10 and 30 mg/kg, SAR441566 is a potent TNF inhibitor *in vivo* and is anti-arthritic in this clinically relevant model of RA. Blood samples taken across studies indicate that these antibody-like efficacious doses were associated with a free average exposure of 24 nM at 10 mg/kg. The *in vivo* efficacy of SAR441566 in all three independent CIA studies is shown in Supplementary Figure S2A–F.

### 3D volumetric micro-CT phenotyping confirms antibody-like inhibition of arthritis development

**FIGURE 4:**
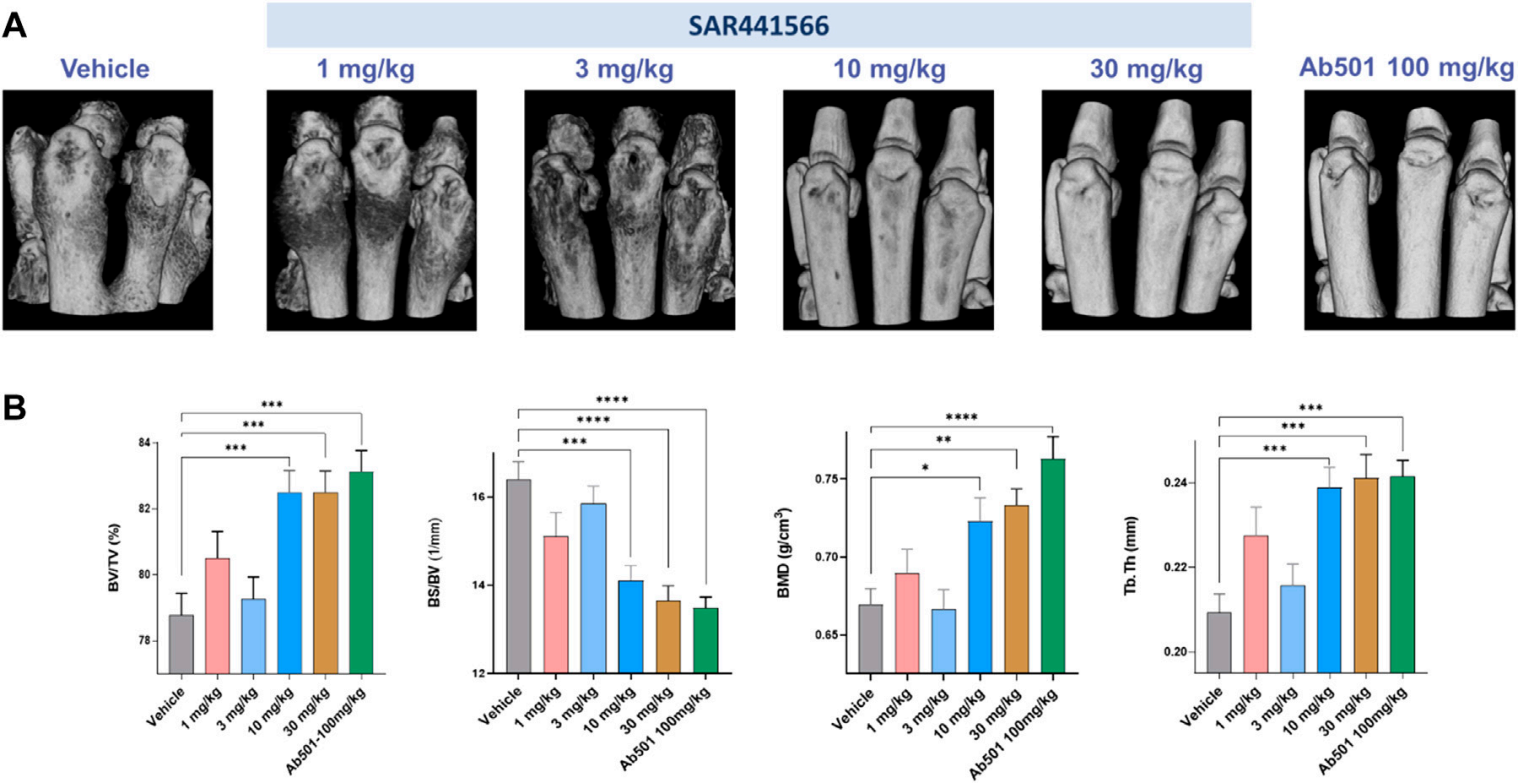
Effect of SAR441566 on bone in the CIA model as assessed by 3D volumetric micro-computed tomography: **(A)** shows a representative 3D rendering view of hind paws from animals treated therapeutically with SAR441566. A dose-dependent trend for bone improvement with SAR441566 is observed and at a dose of 10 and 30 mg/kg these effects are comparable to those seen in the Ab501 treated group. **(B)** 3D volumetric analysis of whole bone microstructure, using the trabecular bone analysis methods and parameters, of the hind paws with a dose-dependent trend for SAR441566 on bone volume fraction (BV/TV), bone surface density (BS/BV), bone mineral density (BMD), and bone fragment thickness (Tb.Th) reaching anti-TNF biologic-like effects at 10 and 30 mg/kg, respectively. Statistical analysis was performed using the one-way ANOVA and Dunnett’s multiple comparisons test, * for *p* < 0.05; ** for *p* < 0.005; *** for *p* < 0.001; and **** for *p* < 0.0001.

Micro-CT imaging, especially high-resolution micro-CT image-based 3D rendering, and interactive visualization of volumetric data from CIA mice limb bones demonstrated typical osteoimmunological lesions in the limbs, such as joint bone erosion, bone destruction, and osteophytes developed on the bone surface. As shown in Figure 4, there is clear evidence that SAR441566 protects against CIA associated bone destruction in a dose dependent manner. Pivotal bone microarchitecture parameters of the hind-paws, such as bone volume fraction (BV/TV, %) were significantly higher in the 10 mg/kg and 30 mg/kg SAR441566 treatment groups as compared to the vehicle group and this effect was also comparable to that seen in Ab501 (100 mg/kg) treated mice (Figure 4B). This micro-CT analysis was conducted on limb bones from one of the three CIA studies where SAR441566 also showed antibody-like inhibition against arthritis development (Supplementary Figures S2E,F).

### Quantitative systems pharmacology model predictions on tumor necrosis factor reduction in synovial tissue

**FIGURE 5:**
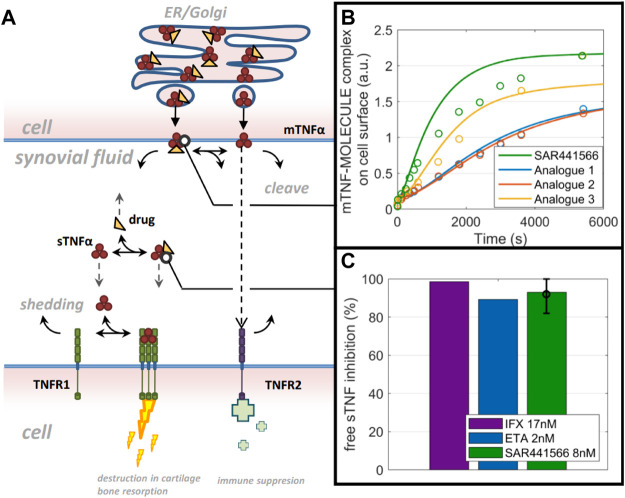
QSP model predictions: A computer model and simulations for synovial TNF biology. **(A)** Reduced model scheme of TNF life cycle and drug binding. **(B)** data on mTNF-SAR441566 complex appearance in comparison to 3 analogues from the same chemical family on cell surface (circles) and model prediction (lines) using molecule specific binding kinetics and intracellular accumulation factors. **(C)** Model prediction on free soluble TNF inhibition in synovial fluid for Infliximab (IFX), Etanercept (ETA) and SAR441566.

Based on the developed TNF model for synovial tissue (reduced model schematic is shown in Figure 5A), free TNF levels in human synovial tissue start with 0.3 p.m. (total TNF: 1.5 p.m.) concentration and decline to very low concentrations (1–10%) during infliximab (3 mg/kg) and etanercept (25 mg) treatment (Figure 5C). To account for the lower drug concentration in synovial fluid compared to blood (Simkin and Bassett, 2011), we used one third of the infliximab and etanercept steady state concentration reported by Tani et al. (2013) as C_ss_^f^ (ETA: 2.2 nM, IFX: 17 nM) in our model. For SAR441566, intracellular binding is assumed to solely depend on drug concentration and the binding kinetics that are equivalent to extracellular TNF binding. To understand the rate of intracellular binding of TNF by SAR441566, the intracellular pre-binding assay was used to measure the appearance of newly formed TNF-compound complex on the cell surface (Figure 5B). Compared to similar compounds of the same chemical series the complex formation was fastest for SAR441566. The model reflects the data under the assumption of equal binding kinetics (k_a_ and k_d_) inside and outside of the cell but taking into account accumulation factors that are specific for each compound. An accumulation factor of 50 explained most of the data for SAR441566 while other reference compounds had higher binding kinetics but lower estimated accumulation factors and, overall, slower TNF-compound complex appearance rates on the cell surface. An accumulation factor of 50 is between the tissue to plasma ratios (Table 4) and ADME calculations of >150-fold (see previous section). With an intracellular accumulation factor of 50 and the slow off rate of SAR441566 from TNF shown in Table 1, the model estimates a reduction in soluble free TNF to less than 10% for a free average concentration of 8 nM (Figure 5C) and 2% for 24 nM (not shown), which is consistent with the estimated occupancy of 90% in the *in vitro* assay at 16 nM and the efficacious free exposure of 24 nM in the CIA mouse model.

### Human PK prediction for SAR441566

Preclinical PK parameters were analyzed in rat, dog and cynomolgus monkey (cyno) after IV and PO dosing (Table 5) and were in accordance with ADME properties described (data summarized in Table 2). A dose proportional increase in exposure was observed in dog and cyno especially at lower doses up to 20–30 mg/kg. The clearance after i.v. application was low to moderate; volume of distribution was high, and bioavailability was moderate to high across species (Tables 2, 5). Using simple three-species allometry, the total human i.v. clearance and V_ss_ were predicted to be 0.40 L/h/kg and 6.4 L/kg, respectively. The human hepatic clearance calculated by in vitro-in vivo extrapolation with and without correction for albumin binding was 0.42 L/h/kg and 0.81 L/h/kg, respectively. The mechanistically modeled V_ss_ value was 5.1 L/kg. Overall, the estimated values for distribution and clearance in human from preclinical data were in line with the prediction using mechanistic approaches based on *in vitro* data and physicochemical properties and should support once a day (QD) or BID dosing in human.

## Discussion

We have previously described the discovery of specific small molecule inhibitors of TNF that stabilize an asymmetrical form of the soluble TNF trimer, compromising downstream signalling and inhibiting TNF function both *in vitro* and *in vivo* (O’Connell et al., 2019). These early molecules, whilst being good tools, did not possess the properties to make them suitable candidates for human therapy. Directly arising from this work, we now identify SAR441566 as a small molecule TNF inhibitor that has the preclinical attributes to become a first in class oral drug with the potential to transform the lives of patients living with multiple TNF driven immune diseases.

The treatment of patients suffering from chronic autoimmune diseases such as RA has been revolutionized over the past two decades by the development and approval of disease modifying anti-TNF biologics which have changed the clinical outcome for millions of patients and has validated TNF as an important therapeutic target.

Despite the overall success of the anti-TNF biologics there remains a significant unmet need in diseases like RA. For example, it has recently been reported that only 26% of RA patients are satisfied with their treatment which includes those on biologic DMARDS (Radawski et al., 2019) and the majority feel that their current therapy is inadequate in controlling their disease (Taylor et al., 2021). In addition, there is a ‘ceiling effect’ reported with biologics, including anti-TNFs, where treatment does not induce disease remission and low disease activity (LDA) in the majority of patients (Smolen and Aletaha, 2015). Some of this ‘ceiling effect’ can be attributed to the immunogenic response that a biologic elicits in patients. It is well documented that biologics can induce an immune response in patients with the generation of anti-drug-antibodies (ADA). This is a common feature and limitation of this class of therapeutic and although this response is variable between patients it is a frequent occurrence at the group level and has been reported across multiple diseases for anti-TNF biologics such as adalimumab, infliximab and biosimilars (Kalden and Schulze-Koops, 2017; Strand et al., 2021). The generation of an ADA response has a range of potential consequences for the patient, from no clinical effect to reduced therapeutic efficacy, injection/infusion site reactions and rare but severe adverse events such as anaphylaxis (Smith et al., 2016; Strand et al., 2021).

The reduced therapeutic effect has been well documented in RA patients and after an initially good response to an anti-TNF biologic efficacy is lost over time leading to secondary drug failure. This loss of efficacy is the main reason for discontinuation of anti-TNF therapy and has been attributed to the ADA response. This, along with the ADA related adverse events such as injection/infusion site reactions, means patients often swap from one anti-TNF biologic to another or switch to an alternative biologic such as rituximab. This immunogenicity therefore reduces the overall number of patients that reach a clinically meaningful response with anti-TNF treatment and would account for the lack of disease control in a proportion of patients. A small molecule anti-TNF inhibitor such as SAR441566 would not be expected to elicit an immunogenic response, mitigating the risk of secondary drug failure, whilst maintaining exposure and hence efficacy as well as reducing any biologic related adverse events. This in turn has the potential for patients to reliably control their disease without having to swap or switch treatments.

A number of studies over the years have also assessed patient preference for the route of administration of their RA medication and oral administration is consistently ranked as first choice for the majority of patients (Barclay et al., 2013; Alten et al., 2016; Taylor et al., 2020). It has also been reported that up to 83% of RA patients who might need to change therapies, as recommended by their doctor, would choose to switch to an oral tablet over an injectable or intravenous infusion (Barclay et al., 2013). In this respect, speed and ease of administration have been highlighted as common reasons for patients’ preference towards oral medication (Taylor et al., 2020). A small molecule such as SAR441566 would therefore address this patient unmet need.

We have demonstrated that SAR441566 binds to TNF with a high affinity (Figure 1A) and importantly that it is potent in an endogenous human TNF dependent whole blood assay. The development of CA 1974, a conformation specific antibody capable of detecting small molecule bound human TNF, has revealed that SAR441566 can bind to intracellular TNF. Furthermore, whilst we had previously described an ELISA using this novel antibody for the specific detection of compound bound TNF (Lightwood et al., 2021), this is the first description of this method used in combination with an ELISA for total TNF to quantify small molecule-mediated occupancy of TNF with the potential to measure target occupancy in clinical studies.

Given the historic use of the *in vivo* CIA model in drug discovery to successfully identify clinically relevant anti-arthritic drugs, which include anti-TNF biologics (Piquet et al., 1992; Bendele et al., 1999; Wang et al., 2013; Dowty et al., 2014) it was key for us to demonstrate efficacy with SAR441566 in this model. Encouragingly, the strong *in vitro* potency profile of SAR441566 was replicated *in vivo* with a dose dependent inhibition in signs of arthritis. Statistical analysis of this data highlighted 10 mg/kg BID as a potent therapeutic dose. Analysis of the hind limbs by micro-CT revealed an attenuation of bone destruction was achieved with SAR441566 at a dose of 10 and 30 mg/kg BID which was in line with that seen in mice treated with the murine anti-TNF antibody (Ab501). The 3D images clearly show a visual difference between SAR441566, and vehicle treated groups (Figure 4A). Furthermore, the 3D micro-CT imaging based volumetric measurements of bone surface and microarchitecture validated the significance, showing an increased BV/TV with SAR441566 treatment which indicates a reduction in bone erosion and destruction; and reduced BS/BV indicating more smooth bone surface due to reduced osteophyte formation.

For human dose predictions, we routinely apply other methods to complement *in vitro* and *in vivo* data, so we used a quantitative systems pharmacology approach (QSP) to assess the potential efficacious concentration of SAR441566. QSP predicted that a reduction in free soluble TNF in synovial tissue to less than 10% would be achieved with SAR441566 at a free C_av_ of 8 nM and this was comparable to data for infliximab and etanercept. This suggests that SAR441566 is likely to have antibody like potency in the clinic at this exposure.

In summary, to determine suitable doses for human trials we generated efficacy data on SAR441566 using a multi-system approach across *in vitro*, *in vivo* and QSP systems. Given the complexities and variabilities of these individual methods it is extremely encouraging that the target efficacy is comparable across all systems, giving a relatively narrow predicted target range of 8–24 nM free SAR441566 C_av_.

SAR441566 possesses the drug like properties to make it suitable for therapeutic use. It has good permeability, low metabolic clearance, high tissue distribution, high fraction unbound, moderate to high bioavailability and accumulates intracellularly where it can pre-bind TNF (Figure 2) prior to surface expression and cleavage. This is important because the slow association rate of this class of molecule would make doses unrealistically high if the low levels of circulating TNF and rapidly turned over extracellular membrane TNF were the only accessible forms of TNF for SAR441566. These properties make it an ideal candidate for inhibiting TNF in blood and tissue. Human PK prediction employing both empirical and mechanistic approaches resulted in similar estimated values for human clearance and volume of distribution, substantiating the consistent PK properties of this compound. Based on all the preclinical data we have generated, SAR441566 is anticipated to show good bioavailability, high tissue distribution and moderate clearance in humans, indicating the potential of once daily oral administration.

The search for a small molecule that could do the same as a biologic in diseases like RA has been a goal for many years. Small molecules not only have an advantage in being administered orally but are expected to have better tissue penetration and can be chemically engineered to enter compartments that protein-based drugs traditionally cannot access. Having described small molecules that block TNF by the unusual mode of stabilizing it in an inactive conformation, we now show the outcome of those initial studies. The resulting molecule, SAR441566, has the potential to be more accessible to patients in the pre-biologic treatment space or after first line drug failure when a swift change in disease management is required. We expect it to deliver a more stable treatment option with no immunogenicity, a reliable exposure and efficacy profile, a reduced propensity to require a swap or switch of medication, as well as the potential elimination of ADA related adverse events whilst still maintaining anti-TNF biologic like disease modifying activity. With these characteristics, SAR441566 has the potential to become the anti-TNF of choice in the pre-biologic phase of treatment and is currently advancing in clinical trials.

## Data Availability

The raw data supporting the conclusions of this article will be made available by the authors, without undue reservation.

## References

[B1] Alexiou P. Papakyriakou A. Ntougkos E. Papaneophytou C. P. Liepouri F. Mettou A. (2014). Rationally designed less toxic SPD-304 analogs and preliminary evaluation of their TNF inhibitory effects. Arch. Pharm. 347 (11), 798–805. 10.1002/ardp.201400198 25160057

[B2] Alexopoulou L. Pasparakis M. Kollias G. (1997). A murine transmembrane tumor necrosis factor (TNF) transgene induces arthritis by cooperative p55/p75 TNF receptor signaling. Eur. J. Immunol. 27, 2588–2592. 10.1002/eji.1830271018 9368614

[B3] Almutairi K. Nossent J. Preen D. Keen H. Inderjeeth C. (2021). The prevalence of rheumatoid arthritis: A systematic review of population-based studies. J. Rheumatol. 48 (5), 669–676. 10.3899/jrheum.200367 33060323

[B4] Alten R. Krüger K. Rellecke J. Schiffner-Rohe J. Behmer O. Schiffhorst G. (2016). Examining patient preferences in the treatment of rheumatoid arthritis using a discrete-choice approach. Patient prefer. Adherence 10, 2217–2228. 10.2147/PPA.S117774 27843301 PMC5098563

[B5] Apostolaki M. Armaka M. Victoratos P. Kollias G. (2010). Cellular mechanisms of TNF function in models of inflammation and autoimmunity. Curr. Dir. Autoimmun. 11, 1–26. 10.1159/000289195 20173385

[B6] Barclay N. Tarallo M. Hendrikx T. Marett S. (2013). Patient preference for oral versus injectable and intravenous methods of treatment for rheumatoid arthritis. Value Health 16, A568. 10.1016/j.jval.2013.08.1521

[B7] Bendele A. McComb J. Gould T. McAbee T. Sennello G. Chlipala E. (1999). Animal models of arthritis: Relevance to human disease. Toxicol. Pathol. 27, 134–142. 10.1177/019262339902700125 10367688

[B8] Black R. A. Rauch C. T. Kozlosky C. J. Peschon J. J. Slack J. L. Wolfson M. F. (1997). A metalloproteinase disintegrin that releases tumour-necrosis factor-α from cells. Nature 385, 729–733. 10.1038/385729a0 9034190

[B9] Brand D. D. Latham K. A. Rosloniec E. F. (2007). Collagen-induced arthritis.Nat. Nat. Protoc. 2 (5), 1269–1275. 10.1038/nprot.2007.173 17546023

[B10] Brockhaus M. Schoenfeld H. J. Schlaeger E. J. Hunziker W. Lesslauer W. Loetscher H. (1990). Identification of two types of tumor necrosis factor receptors on human cell lines by monoclonal antibodies. Proc. Natl. Acad. Sci. U. S. A. 87 (8), 3127–3131. 10.1073/pnas.87.8.3127 2158104 PMC53847

[B11] Chan F. K. Chun H. J. Zheng L. Siegel R. M. Bui K. L. Lenardo M. J. (2000). A domain in TNF receptors that mediates ligand-independent receptor assembly and signaling. Science 288, 2351–2354. 10.1126/science.288.5475.2351 10875917

[B12] Chappard D. Legrand E. Haettich B. Chalès G. Auvinet B. Eschard J. P. (2001). Fractal dimension of trabecular bone: Comparison of three histomorphometric computed techniques for measuring the architectural two-dimensional complexity. J. Pathol. 195, 515–521. 10.1002/path.970 11745685

[B13] Chen X. Oppenheim J. J. (2011). Contrasting effects of TNF and anti-TNF on the activation of effector T cells and regulatory T cells in autoimmunity. FEBS Lett. 585 (23), 3611–3618. 10.1016/j.febslet.2011.04.025 21513711 PMC3164898

[B14] Chen X. Wu X. Zhou Q. Howard O. M. Z. Netea M. G. Oppenheim J. J. (2013). TNFR2 is critical for the stabilization of the CD4+Foxp3+ regulatory T cell phenotype in the inflammatory environment. J. Immunol. 190 (3), 1076–1084. 10.4049/jimmunol.1202659 23277487 PMC3552130

[B15] Cooles F. A. H. Isaacs J. D. (2011). Pathophysiology of rheumatoid arthritis. Curr. Opin. Rheumatol. 23 (3), 233–240. 10.1097/BOR.0b013e32834518a3 21427580

[B16] Cope A. P. Aderka D. Doherty M. Engelmann H. Gibbons D. Jones A. C. (1992). Increased levels of soluble tumor necrosis factor receptors in the sera and synovial fluid of patients with rheumatic diseases. Arthritis Rheum. 35 (10), 1160–1169. 10.1002/art.1780351008 1329774

[B17] Deng G. Zheng L. Chan F. K. Lenardo M. (2005). Amelioration of inflammatory arthritis by targeting the pre-ligand assembly domain of tumor necrosis factor receptors. Nat. Med. 11, 1066–1072. 10.1038/nm1304 16170321

[B18] Deng G. Zheng L. Tsokos G. (2010). Targeted tumor necrosis factor receptor I preligand assembly domain improves skin lesions in MRL/lpr mice. Arthritis Rheum. 62 (8), 2424–2431. 10.1002/art.27534 20506390 PMC2921998

[B19] Deng X. Zhang X. Tang B. Liu H. Shen Q. Liu Y. (2018). Design, synthesis, and evaluation of dihydrobenzo[cd]indole-6-sulfonamide as TNF-α inhibitors. Front. Chem. 4 (6), 98. 10.3389/fchem.2018.00098 PMC5893771 29670876

[B20] Dietrich J. D. Longenecker K. L. Wilson N. S. Goess C. Panchal S. C. Swann S. L. (2021). Development of orally efficacious allosteric inhibitors of TNFα via fragment-based drug design. J. Med. Chem. 64, 417–429. 10.1021/acs.jmedchem.0c01280 33378180

[B21] Dömling A. Xin L. (2021). TNF-α: The shape of small molecules to come? Drug Discov. Today 27 (1), 3–7. 10.1016/j.drudis.2021.06.018 34229081

[B22] Dowty M. E. Jesson M. I. Ghosh S. Lee J. Meyer D. M. Krishnaswami S. (2014). Preclinical to clinical translation of tofacitinib, a Janus kinase inhibitor, in rheumatoid arthritis. J. Pharmacol. Exp. Ther. 348 (1), 165–173. 10.1124/jpet.113.209304 24218541

[B23] Edmunds J. J. (2019). “Untangling perception and reality: Small-molecule TNF-α inhibitors,” in Medicinal chemistry reviews. Ref. n°: REV2019-ch6. Editor Bronson J. J. (Boston, MA: Medicinal Chemistry Division of the American Chemical Society).

[B24] Fallahi-Sichani M. El-Kebir M. Marino S. Kirschner D. E. Linderman J. J. (2011). Multiscale computational modeling reveals a critical role for TNF-α receptor 1 dynamics in tuberculosis granuloma formation. J. Immunol. 15 (6), 3472–3483. 10.4049/jimmunol.1003299 PMC3127549 21321109

[B25] Fong Y. Tracey K. J. Moldawer L. L. Hesse D. G. Manogue K. B. Kenney J. S. (1989). Antibodies to cachectin/tumor necrosis factor reduce interleukin 1 beta and interleukin 6 appearance during lethal bacteremia. J. Exp. Med. 170 (5), 1627–1633. 10.1084/jem.170.5.1627 2809510 PMC2189514

[B26] Gelman A. Carlin J. Stern H. Rubin D. (2004). Bayesian data analysis. 2nd Edn. (Boca Raton, FL: CRC Press/Chapman & Hall).

[B27] Gough P. Myles I. (2020). Tumor necrosis factor receptors: Pleiotropic signaling complexes and their differential effects. Front. Immunol. 11, 585880. 10.3389/fimmu.2020.585880 33324405 PMC7723893

[B28] Grell M. Douni E. Wajant H. Löhden M. Clauss M. Maxeiner B. (1995). The transmembrane form of tumor necrosis factor is the prime activating ligand of the 80 kDa tumor necrosis factor receptor. Cell 83 (5), 793–802. 10.1016/0092-8674(95)90192-2 8521496

[B29] Grell M. Wajant H. Zimmermann G. Scheurich P. (1998). The type 1 receptor (CD120a) is the high-affinity receptor for soluble tumor necrosis factor. Proc. Natl. Acad. Sci. U. S. A. 95 (2), 570–575. 10.1073/pnas.95.2.570 9435233 PMC18461

[B30] He M. M. Smith A. S. Oslob J. D. Flanagan W. M. Braisted A. C. Whitty A. (2005). Small-molecule inhibition of TNF-alpha. Science 310, 1022–1025. 10.1126/science.1116304 16284179

[B31] Hohmann H. P. Brockhaus M. Baeuerle P. A. Remy R. Kolbeck R. Van Loon A. P. (1990). Expression of the types A and B tumor necrosis factor (TNF) receptors is independently regulated, and both receptors mediate activation of the transcription factor NF-kappa B. TNF alpha is not needed for induction of a biological effect via TNF receptors. J. Biol. Chem. 265, 22409–22417. 10.1016/s0021-9258(18)45720-1 2176217

[B32] Holt K. Ye M. Nagar S. Korzekwa K. (2019). Prediction of tissue-plasma partition coefficients using microsomal partitioning: Incorporation into physiologically based pharmacokinetic models and steady-state volume of distribution predictions. Drug Metab. Dispos. 47 (10), 1050–1060. 10.1124/dmd.119.087973 31324699 PMC6750188

[B33] Jang D. Lee A. Shin H.-Y. Song H.-R. Park J.-H. Kang T.-B. (2021). The role of tumor necrosis factor Alpha (TNF-α) in autoimmune disease and current TNF-α inhibitors in therapeutics. Int. J. Mol. Sci. 22, 2719. 10.3390/ijms22052719 33800290 PMC7962638

[B34] Johnson K. J. Sanchez H. N. Schoenbrunner N. (2019). Defining response to TNF-inhibitors in rheumatoid arthritis: The negative impact of anti-TNF cycling and the need for a personalized medicine approach to identify primary non-responders. Clin. Rheumatol. 38, 2967–2976. 10.1007/s10067-019-04684-1 31520227

[B35] Jones E. Y. Stuart D. I. Walker N. P. C. (1989). Structure of tumour necrosis factor. Nature 338 (6212), 225–228. 10.1038/338225a0 2922050

[B36] Kalden J. Schulze-Koops H. (2017). Immunogenicity and loss of response to TNF inhibitors: Implications for rheumatoid arthritis treatment. Nat. Rev. Rheumatol. 13, 707–718. 10.1038/nrrheum.2017.187 29158574

[B37] Kalliolias G. D. Ivashkiv L. B. (2016). TNF biology, pathogenic mechanisms and emerging therapeutic strategies. Nat. Rev. Rheumatol. 12, 49–62. 10.1038/nrrheum.2015.169 26656660 PMC4809675

[B38] Lee A. Qiao Y. Grigoriev G. Chen J. Park-Min K. H. Park S. H. (2013). Tumor necrosis factor α induces sustained signaling and a prolonged and unremitting inflammatory response in rheumatoid arthritis synovial fibroblasts. Arthritis Rheum. 65, 928–938. 10.1002/art.37853 23335080 PMC3618592

[B39] Lightwood D. J. Munro R. J. Porter J. McMillan D. Carrington B. Turner A. (2021). A conformation-selective monoclonal antibody against a small molecule-stabilised signalling-deficient form of TNF. Nat. Commun. 12, 583. 10.1038/s41467-020-20825-6 33495445 PMC7835358

[B40] Lipinski C. A. Lombardo F. Dominy B. W. Feeney P. J. (1997). Experimental and computational approaches to estimate solubility and permeability in drug discovery and development settings. Adv. Drug Deliv. Rev. 23 (1–3), 3–26. 10.1016/s0169-409x(00)00129-0 11259830

[B41] Mahmood I. Balian J. D. (1996). Interspecies scaling: A comparative study for the prediction of clearance and volume using two or more than two species. Life Sci. 59 (7), 579–585. 10.1016/0024-3205(96)00339-6 8761347

[B42] Mahmud S. A. Manlove L. S. Schmitz H. M. Xing Y. Wang Y. Owen D. L. (2014). Costimulation via the tumor-necrosis factor receptor superfamily couples TCR signal strength to the thymic differentiation of regulatory T cells. Nat. Immunol. 15 (5), 473–481. 10.1038/ni.2849 24633226 PMC4000541

[B43] McMillan D. Martinez-Fleites C. Porter J. Davis R. Mori P. Ceska T. (2021). Structural insights into the disruption of TNF-TNFR1 signalling by small molecules stabilising a distorted TNF. Nat. Commun. 12, 582. 10.1038/s41467-020-20828-3 33495441 PMC7835368

[B44] McNearney T. Baethge B. A. Cao S. Alam R. Lisse J. R. Westlund K. N. (2004). Excitatory amino acids, TNF-alpha, and chemokine levels in synovial fluids of patients with active arthropathies. Clin. Exp. Immunol. 137 (3), 621–627. 10.1111/j.1365-2249.2004.02563.x 15320917 PMC1809131

[B45] Mehta A. K. Gracias D. T. Croft M. (2018). TNF activity and T cells. Cytokine 101, 14–18. 10.1016/j.cyto.2016.08.003 27531077 PMC5305780

[B46] Mellado M. Martínez-Muñoz L. Cascio G. Lucas P. Pablos J. L. Rodríguez-Frade J. M. (2015). T cell migration in rheumatoid arthritis. Front. Immunol. 6, 384. 10.3389/fimmu.2015.00384 26284069 PMC4515597

[B47] Ménochet K. Kenworthy K. E. Houston J. B. Galetin A. (2012). Use of mechanistic modeling to assess interindividual variability and interspecies differences in active uptake in human and rat hepatocytes. Drug Metab. Dispos. 40 (9), 1744–1756. 10.1124/dmd.112.046193 22665271 PMC3422540

[B48] Mitoma H. Horiuchi T. Tsukamoto H. Uedaet N. (2018). Molecular mechanisms of action of anti-TNF-α agents - comparison among therapeutic TNF-α antagonists. Cytokine 101, 56–63. 10.1016/j.cyto.2016.08.014 27567553

[B49] Monaco C. Nanchahal J. Taylor P. Feldmann M. (2014). Anti-TNF therapy: Past, present and future. Int. Immunol. 27 (1), 55–62. 10.1093/intimm/dxu102 25411043 PMC4279876

[B50] Montecucco F. Steffens S. Burger F. Da Costa A. Bianchi G. Bertolotto M. (2008). Tumor necrosis factor-alpha (TNF-alpha) induces integrin CD11b/CD18 (Mac-1) up-regulation and migration to the CC chemokine CCL3 (MIP-1alpha) on human neutrophils through defined signalling pathways. Cell. Signal. 20 (3), 557–568. 10.1016/j.cellsig.2007.11.008 18164590

[B51] Moss M. Jin S. L. Milla M. Burkhart W. Carter H. L. Chen W. J. (1997). Cloning of a disintegrin metalloproteinase that processes precursor tumour-necrosis factor-alpha. Nature 385, 733–736. 10.1038/385733a0 9034191

[B52] O’Connell J. Porter J. Kroeplien B. Norman T. Rapecki S. Davis R. (2019). Small molecules that inhibit TNF signalling by stabilising an asymmetric form of the trimer. Nat. Commun. 10, 5795. 10.1038/s41467-019-13616-1 31857588 PMC6923382

[B53] Papadopoulou D. Drakopoulos A. Lagarias P. Melagraki G. Kollias G. Afantitis A. (2021). *In silico* identification and evaluation of natural products as potential Tumor Necrosis Factor Function inhibitors using advanced Enalos Asclepios KNIME nodes. Int. J. Mol. Sci. 22, 10220. 10.3390/ijms221910220 34638561 PMC8508374

[B54] Papaneophytou C. Alexiou P. Papakyriakou A. Ntougkos E. Tsiliouka K. Maranti A. (2015). Synthesis and biological evaluation of potential small molecule inhibitors of tumor necrosis factor. Medchemcomm 6 (6), 1196–1209. 10.1039/c5md00023h

[B55] Piquet P. F. Grau G. E. Vesin C. Loetscher H. Gentz R. Lesslauer W. (1992). Evolution of collagen arthritis in mice is arrested by treatment with anti-tumour necrosis factor (TNF) antibody or a recombinant soluble TNF receptor. Immunology 77, 510–514. 1337334 PMC1421669

[B56] Poulin P. Kenny J. R. Hop C. E. C. A. Haddad S. (2012). *In vitro*-*in vivo* extrapolation of clearance: Modeling hepatic metabolic clearance of highly bound drugs and comparative assessment with existing calculation methods. J. Pharm. Sci. 101 (2), 838–851. 10.1002/jps.22792 22009717

[B57] Radawski C. Genovese M. C. Hauber B. Nowell W. B. Hollis K. Gaich C. L. (2019). Patient perceptions of unmet medical need in rheumatoid arthritis: A cross-sectional survey in the USA. Rheumatol. Ther. 6 (3), 461–471. 10.1007/s40744-019-00168-5 31385264 PMC6702617

[B58] Rodgers T. Rowland M. (2007). Mechanistic approaches to volume of distribution predictions: Understanding the processes. Pharm. Res. 24 (5), 918–933. 10.1007/s11095-006-9210-3 17372687

[B59] Salomon B. L. (2021). Insights into the biology and therapeutic implications of TNF and regulatory T cells. Nat. Rev. Rheumatol. 17, 487–504. 10.1038/s41584-021-00639-6 34226727

[B60] Sanguedolce M. V. Capo C. Bongrand P. Mege J. L. (1992). Zymosan-stimulated tumor necrosis factor-alpha production by human monocytes. Down-modulation by phorbol ester. J. Immunol. 148 (7), 2229–2236. 1347552

[B61] Schmitt M. V. Lienau P. Fricker G. Reichel A. (2019). Quantitation of lysosomal trapping of basic lipophilic compounds using *in vitro* assays and in silico predictions based on the determination of the full pH profile of the endo-/lysosomal system in rat hepatocytes. Drug Metab. Dispos. 47 (1), 49–57. 10.1124/dmd.118.084541 30409837

[B62] Shazeeb M. S. Cox M. K. Gupta A. Tang W. Singh K. Pryce C. T. (2018). Skeletal characterization of the Fgfr3 mouse model of achondroplasia using micro-CT and MRI volumetric imaging. Sci. Rep. 8 (1), 469. 10.1038/s41598-017-18801-0 29323153 PMC5765052

[B63] Shurety W. Merino-Trigo A. Brown D. Hume D. A. Stow J. L. (2000). Localization and post-Golgi trafficking of tumor necrosis factor-alpha in macrophages. J. Interferon Cytokine Res. 20 (4), 427–438. 10.1089/107999000312379 10805378

[B64] Simkin P. A. Bassett J. E. (2011). Pathways of microvascular permeability in the synovium of normal and diseased human knees. J. Rheumatol. 38, 2635–2642. 10.3899/jrheum.110785 22045843

[B65] Skyscan (2009). Structural parameters measured by SkyScan CTanalyzer software, 36p. Belgium. Available at: https://www.microphotonics.com/wp-content/uploads/2016/01/CTAn_parameters.pdf .

[B66] Smith A. Manoli H. Jaw S. Frutoz K. Epstein A. L. Khawli L. A. (2016). Unraveling the effect of immunogenicity on the PK/PD, efficacy, and safety of therapeutic proteins. J. Immunol. Res. 2016, 2342187. 10.1155/2016/2342187 27579329 PMC4992793

[B67] Smolen J. S. Aletaha D. Barton A. Burmester G. R. Emery P. Firestein G. S. (2018). Rheumatoid arthritis. Nat. Rev. Dis. Prim. 4, 18001. 10.1038/nrdp.2018.1 29417936

[B68] Smolen J. S. Aletaha D. (2015). Rheumatoid arthritis therapy reappraisal: Strategies, opportunities and challenges. Nat. Rev. Rheumatol. 11, 276–289. 10.1038/nrrheum.2015.8 25687177

[B69] Smolen J. S. Aletaha D. McInnes I. B. (2016). Rheumatoid arthritis. Lancet 388, 2023–2038. 10.1016/S0140-6736(16)30173-8 27156434

[B70] Strand V. Goncalves J. Isaacs J. D. (2021). Immunogenicity of biologic agents in rheumatology. Nat. Rev. Rheumatol. 17, 81–97. 10.1038/s41584-020-00540-8 33318665

[B71] Sun W. Wu Y. Zheng M. Yang Y. Liu Y. Wu C. (2020). Discovery of an orally active small-molecule tumor necrosis factor-α inhibitor. J. Med. Chem. 63, 8146–8156. 10.1021/acs.jmedchem.0c00377 32667202

[B72] Tani K. Takayanagi R. Yokoyama H. Yamada Y. (2013). Theoretical analysis of efficacy of biological agent for rheumatoid arthritis based on target molecular binding occupancy. Rheumatol. Int. 33 (7), 1791–1795. 10.1007/s00296-012-2650-7 23300004

[B73] Tartaglia L. A. Pennica D. Goeddel D. V. (1993). Ligand passing: The 75-kDa tumor necrosis factor (TNF) receptor recruits TNF for signaling by the 55-kDa TNF receptor. J. Biol. Chem. 268, 18542–18548. 10.1016/s0021-9258(17)46661-0 8395508

[B74] Taylor P. C. Betteridge N. Brown T. M. Woolcott, J Kivitz A. J. Zerbini C. (2020). Treatment mode preferences in rheumatoid arthritis: Moving toward shared decision-making. Patient prefer. Adherence 14, 119–131. 10.2147/PPA.S220714 32021123 PMC6980841

[B75] Taylor P. C. Woods M. Rycroft C. Patel P. Blanthorn-Hazell S. Kent T. (2021). Targeted literature review of current treatments and unmet need in moderate rheumatoid arthritis in the United Kingdom. Rheumatology 60 (11), 4972–4981. 10.1093/rheumatology/keab464 34080612 PMC8566217

[B76] Vassalli P. (1992). The pathophysiology of tumor necrosis factors. Annu. Rev. Immunol. 10, 411–452. 10.1146/annurev.iy.10.040192.002211 1590993

[B77] Walley R. Sherington J. Rastrick J. Detrait E. Hanon E. Watt G. (2016). Using Bayesian analysis in repeated preclinical *in vivo* studies for a more effective use of animals. Pharm. Stat. 15 (3), 277–285. 10.1002/pst.1748 27028721

[B78] Wang Q. T. Wu Y. J. Huang B. Ma Y. K. Song S. S. Zhang L. L. (2013). Etanercept attenuates collagen-induced arthritis by modulating the association between BAFFR expression and the production of splenic memory B cells. Pharmacol. Res. 68 (1), 38–45. 10.1016/j.phrs.2012.11.003 23178558

[B79] Wang S. Shi X. Li J. Huang Q. Ji Q. Wang T. (2022). A small molecule selected from a DNA-encoded library of natural products that binds to TNF-α and attenuates inflammation *in vivo* . Adv. Sci. 9 (21), e2201258. 10.1002/advs.202201258 PMC9313502 35596609

[B80] Ward-Kavanagh L. K. Lin W. W. Šedý J. R. Ware C. F. (2016). The TNF receptor superfamily in Co-stimulating and Co-inhibitory responses. Immunity 44, 1005–1019. 10.1016/j.immuni.2016.04.019 27192566 PMC4882112

[B81] Willrich M. A. Murray D. L. Snyder M. R. (2015). Tumor necrosis factor inhibitors: Clinical utility in autoimmune diseases. Transl. Res. 165, 270–282. 10.1016/j.trsl.2014.09.006 25305470

[B82] Xiao H.-Y. Li N. Duan J. W. J. Jiang B. Lu Z. Ngu K. (2020). Biologic-like *in vivo* efficacy with small molecule inhibitors of TNFα identified using scaffold hopping and structure-based drug design approaches. J. Med. Chem. 63 (23), 15050–15071. 10.1021/acs.jmedchem.0c01732 33261314

[B83] Ying X. Barlow N. J. Feuston M. H. (2017). Chapter 63—micro–computed tomography and volumetric imaging in developmental toxicology in Reproductive and developmental toxicology. 2nd Edition, Editor Gupta R. C. (Massachusetts, United States: Academic Press), 1183–1205. 10.1016/B978-0-12-804239-7.00063-9

[B84] Young S. H. Ye J. Frazer D. G. Shi X. Castranova V. (2001). Molecular mechanism of tumor necrosis factor-alpha production in 1-->3-beta-glucan (zymosan)-activated macrophages. J. Biol. Chem. 276, 20781–20787. 10.1074/jbc.M101111200 11259437

[B85] Zarkesh-Esfahani H. Pockley A. G. Wu Z. Hellewell P. G. Weetman A. P. Ross R. J. (2004). Leptin indirectly activates human neutrophils via induction of TNF-alpha. J. Immunol. 172 (3), 1809–1814. 10.4049/jimmunol.172.3.1809 14734764

[B86] Zunke F. Rose-John S. (2017). The shedding protease ADAM17: Physiology and pathophysiology. Biochim. Biophys. Acta. Mol. Cell Res. 1864, 2059–2070. 10.1016/j.bbamcr.2017.07.001 28705384

