# Corrigendum: An orally available small molecule that targets soluble TNF to deliver anti-TNF biologic-like efficacy in rheumatoid arthritis

**DOI:** 10.3389/fphar.2024.1479557

**Published:** 2024-10-16

**Authors:** Alexander Vugler, James O’connell, Mai Anh Nguyen, Dietmar Weitz, Thomas Leeuw, Elizabeth Hickford, Alexander Verbitsky, Xiaoyou Ying, Markus Rehberg, Bruce Carrington, Mark Merriman, Andrew Moss, Jean-Marie Nicolas, Phil Stanley, Sara Wright, Tim Bourne, Yann Foricher, Zhaoning Zhu, Daniel Brookings, Helen Horsley, Jag Heer, Laurent Schio, Matthias Herrmann, Srinivas Rao, Markus Kohlmann, Peter Florian

**Affiliations:** ^1^ Immunology Therapeutic Area, PV Early Solutions, UCB Pharma, Slough, United Kingdom; ^2^ Discovery Sciences, PV Early Solutions, UCB Pharma, Slough, United Kingdom; ^3^ Sanofi R&D, TMED Pharmacokinetics Dynamics and Metabolism, Frankfurt am Main, Germany; ^4^ Sanofi R&D, Drug Metabolism and Pharmacokinetics, Frankfurt am Main, Germany; ^5^ Sanofi R&D, Type 1/17 Immunology, Immunology and Inflammation Research TA, Frankfurt, Germany; ^6^ Development Science, PV Early Solutions, UCB Pharma, Slough, United Kingdom; ^7^ Sanofi R&D, Translation In vivo Models, Cambridge, MA, United States; ^8^ Sanofi R&D, Translational Disease Modelling, Frankfurt am Main, Germany; ^9^ Translational Medicine Immunology, PV Early Solutions, UCB Pharma, Slough, United Kingdom; ^10^ Development Science, Drug Metabolism and Pharmacokinetics, UCB Pharma, Braine-I’Alleud, Belgium; ^11^ Early PV Missions, PV Early Solutions, UCB Pharma, Slough, United Kingdom; ^12^ Milvuswood Consultancy, Penn, United Kingdom; ^13^ Sanofi R&D, Integrated Drug Discovery, Vitry-sur-Seine, France; ^14^ Global Chemistry, Discovery Sciences, PV Early Solutions, UCB Pharma, Slough, United Kingdom; ^15^ Sanofi R&D, Early Clinical Development, Therapeutic Area Immunology and Inflammation, Frankfurt am Main, Germany

**Keywords:** TNF, rheumatoid arthritis, small molecule, TNF trimer, immunogenicity

In the published article, there was an error in **Affiliation 13**. Instead of “Sanofi R&D, Integrated Drug Discovery, Medicinal Chemistry, Therapeutic Area Immunology & Inflammation, Vitry-sur-Seine, France”, it should be “Sanofi R&D, Integrated Drug Discovery, Vitry-sur-Seine, France”.

In the published article, there was an error in the **Author list**, and authors Zhaoning Zhu, Jag Heer, and Laurent Schio were erroneously excluded. The corrected author list appears below.

Alexander Vugler^1^, James O’Connell^2^*, Mai Anh Nguyen^3^, Dietmar Weitz^4^, Thomas Leeuw^5^, Elizabeth Hickford^6^, Alexander Verbitsky^7^*, Xiaoyou Ying^7^, Markus Rehberg^8^, Bruce Carrington^2^, Mark Merriman^1^, Andrew Moss^9^, Jean-Marie Nicholas^10^, Phil Stanley^1^, Sara Wright^11^, Tim Bourne^12^, Yann Foricher^13^, Zhaoning Zhu^14^, Daniel Brookings^14^, Helen Horsley^14^, Jag Heer^14^, Laurent Schio^13^, Matthias Herrmann^5^, Srinivas Rao^7^, Markus Kohlmann^15^ and Peter Florian^5†^

In the published article, there was an error in the **Conflict of Interest** statement. The individual involvement of Zhaoning Zhu, Jag Heer, and Laurent Schio was omitted. The correct Conflict of Interest statement appears below.

In the published article, there was an error in the **Author contributions** statement. The individual contributions of Zhaoning Zhu, Jag Heer, and Laurent Schio were omitted. The correct Author contributions statement appears below.

