# Supplementary figures and images for: An orally available small molecule that targets soluble TNF to deliver anti-TNF biologic-like efficacy in rheumatoid arthritis
Source: Front Pharmacol. 2022 Nov 16;13:1037983. doi: 10.3389/fphar.2022.1037983 (PMC9709720; doi:10.3389/fphar.2022.1037983)

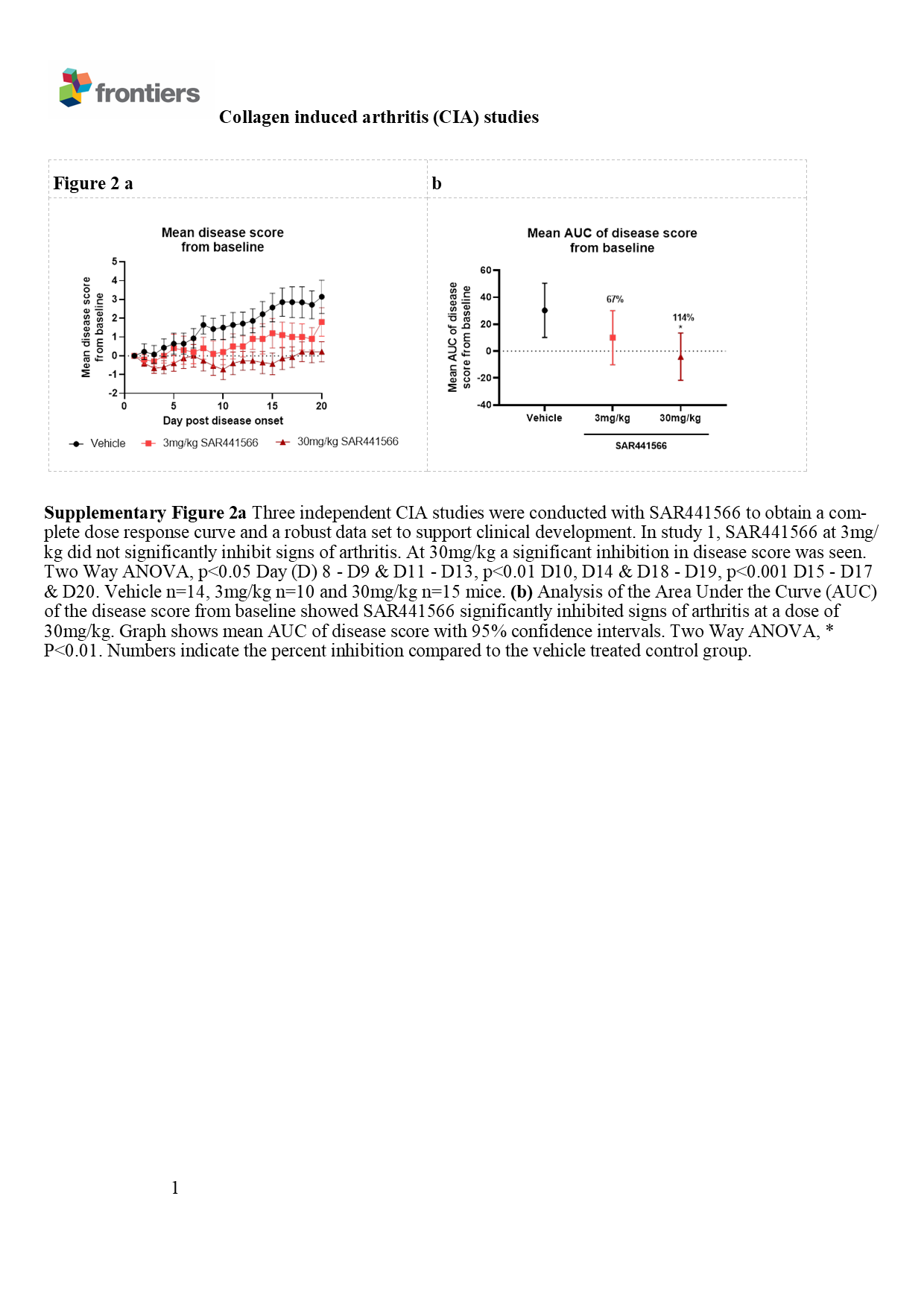

Supplement: Supplementary file 1 [file Image3.tif]

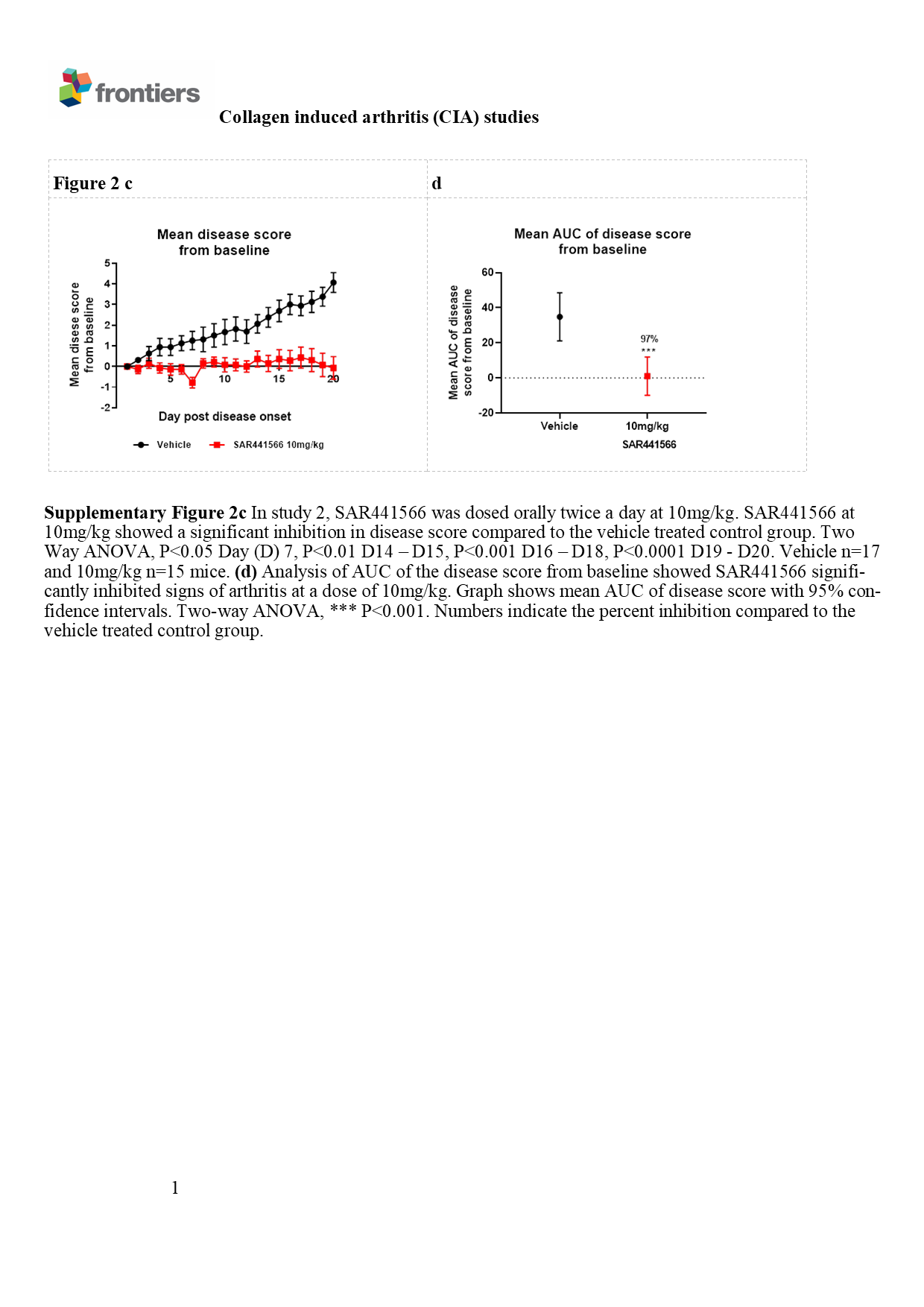

Supplement: Supplementary file 2 [file Image4.tif]

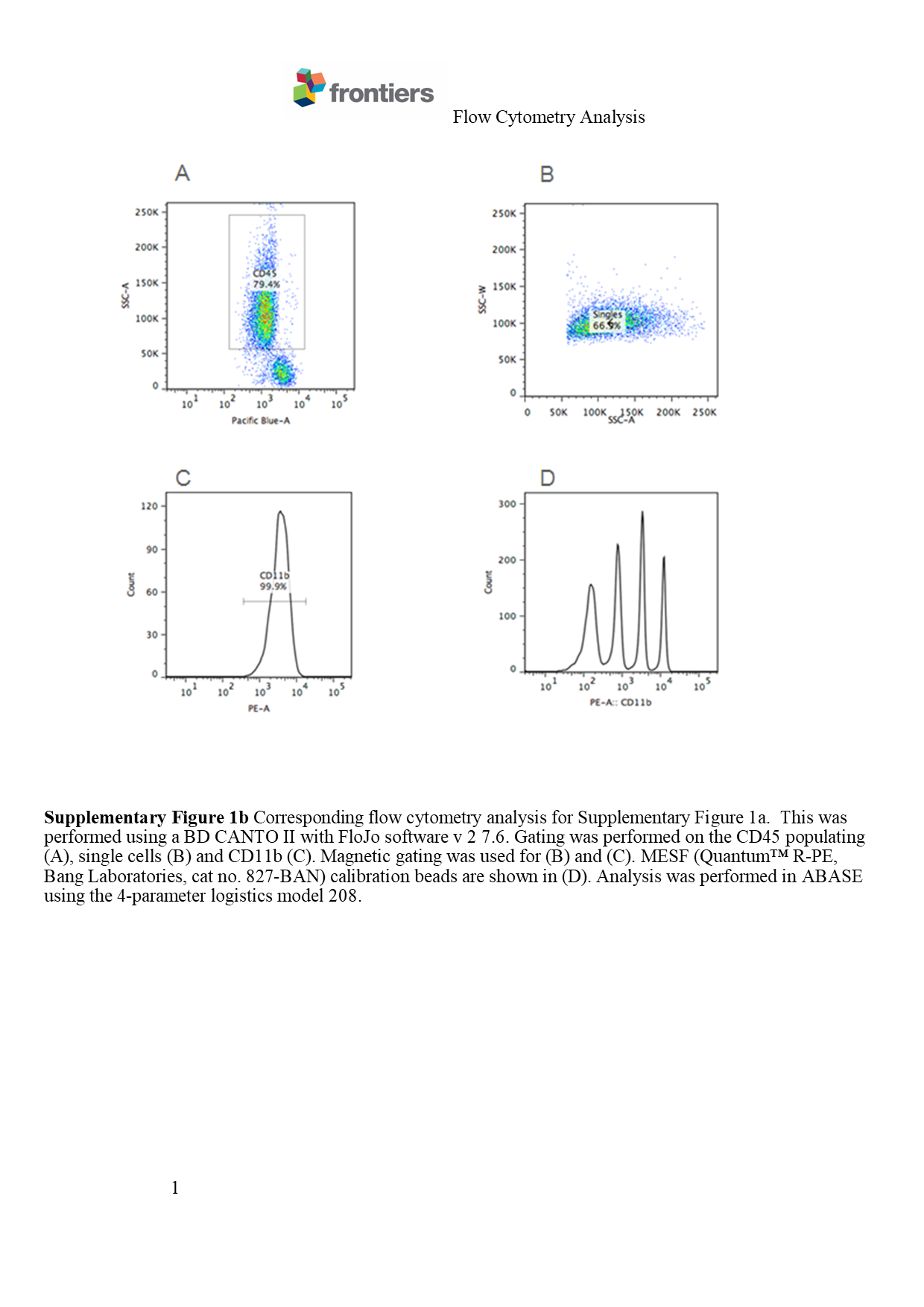

Supplement: Supplementary file 3 [file Image2.tif]

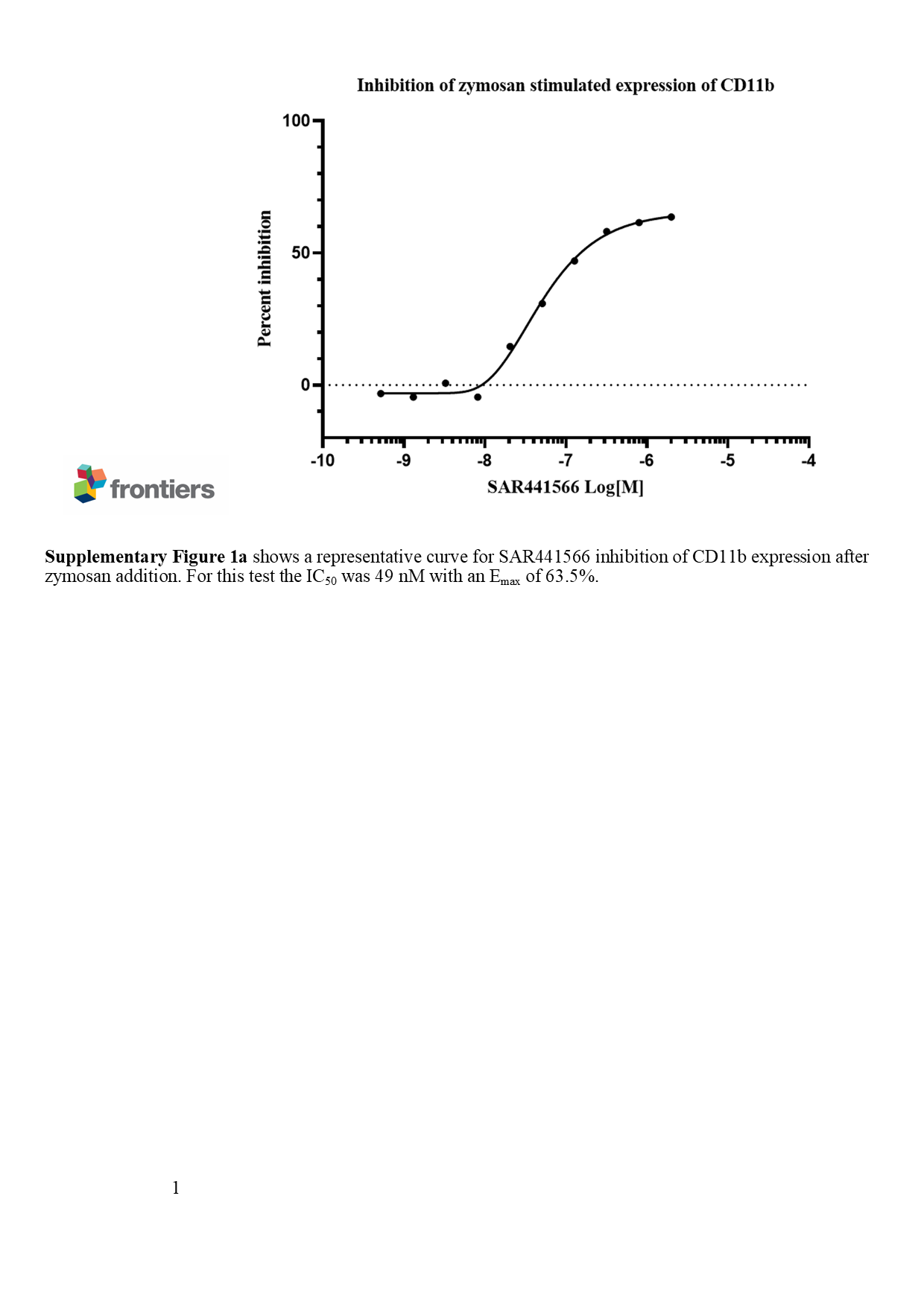

Supplement: Supplementary file 4 [file Image1.TIF]

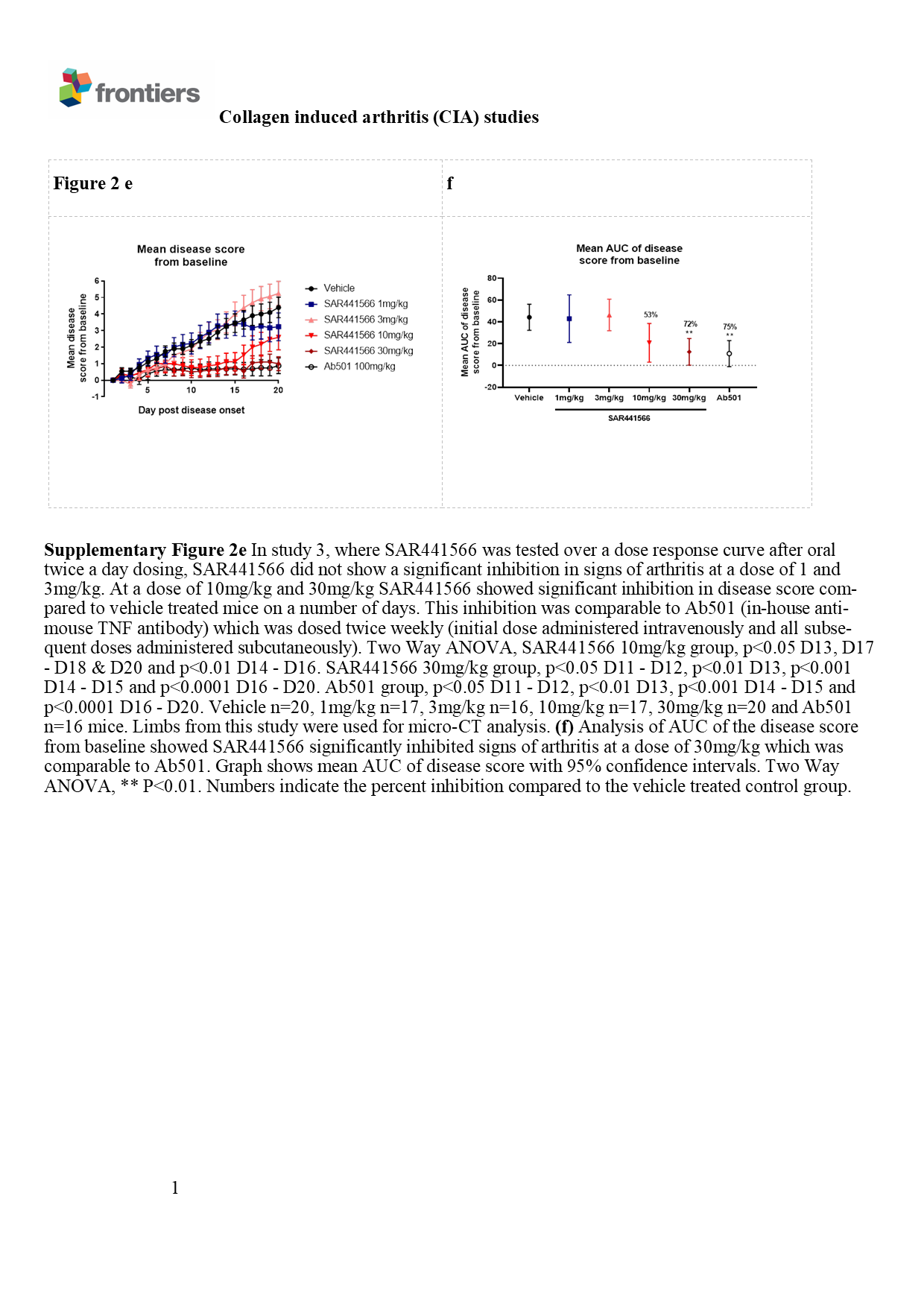

Supplement: Supplementary file 5 [file Image5.tif]
